# Magneto**-**rheological dataset for an extra heavy crude oil (8.5°API) in the presence of a constant magnetic field

**DOI:** 10.1016/j.dib.2019.103902

**Published:** 2019-04-05

**Authors:** Manuel Roa, Ernesto Aguilera, Rodrigo Correa

**Affiliations:** a Escuela de Ingeniería Metalúrgica y Ciencia de los Materiales, Colombia; b Escuela de Ingenierías Eléctrica, Electrónica y de Telecomunicaciones Universidad Industrial de Santander, Colombia

**Keywords:** Magneto-rheology, Heavy crude, Magnetic induction, Viscosity of extra heavy crude

## Abstract

The dataset in this article includes magneto-rheological parameters of an extra heavy Colombian crude oil (8.5°API) in the presence of a static magnetic field. Considering the availability of this type of data is limited, the parameter values for Shear Rate, Shear Stress, Viscosity, Rotational Speed, Torque and Viscosity for three temperatures, i.e., 30, 50 and 70 °C, are hereby included in detail. The shear rate was systematically varied from 1 to 100 [s^−1^]. In the same way, another data set was obtained by applying a magnetic field of 0.17, 0.35 and 0.65 T (this parameter is also known as magnetic flux density or magnetic induction, in Teslas [T]). Enough experimental data for each temperature, were gathered, to generate the magneto-rheological curves for the extra heavy crude oil samples, with and without the presence of a static magnetic field.

**Table:** Specifications table

Subject area	*Chemical Engineering*
More specific subject area	*Magneto-Rheology*
Type of data	*Tables and figures*
How data was acquired	*Anton Paar Rheometer MCR 302 with a magnetic device (for online measurement of magnetic flux density. Measuring system: Parallel plate* 20 mm*. Measuring cell & accessories-magneto-rheological cell MRD 1 T extended)*
Data format	*Raw data*
Experimental factors	*A Rheological properties test was conducted for random selected heavy crude oil samples, at varying temperatures*
Experimental features	*Once the geometry to be used in the magneto-viscometer was selected (cone-plate) the sample was left untouched for a prudential time to homogenize its temperature. The system allowed the automatic definition of the conditions for the experiment, therefore, the collection of experimental data did not require the participation of the user*
Data source location	*Universidad Industrial de Santander, Bucaramanga, Colombia*
Data accessibility	*Data included in this article*
Related research article	*C. Balan, D. Broboana, E. Gheorghiu, L. Vékás* *Rheological characterization of complex fluids in electromagnetic fields, Journal of Non-Newtonian Fluid Mechanics, (154) (1) (2008),pp. 22–30*[1]

**Table:** 

**Value of the data** • These data can contribute to the analysis and comparison of the rheological and magneto-rheological properties of heavy hydrocarbons from other regions. • These experimental data can also be used in the simulation of reservoirs and heavy crude transport, where knowledge of intrinsic magneto-rheological properties is required. • Additionally, these data can support new initiatives for eventual cost reduction methods, improving the efficiency of recovery and refining processes, through the use of crude oil or additive-modified oil intrinsic magneto-rheological properties. • These data can further be used as an initial guide to developing useful models to evaluate the real importance and origin of the magneto-rheological properties of heavy and extra heavy crude oils.

## 1 Data

**Fig. 1:**
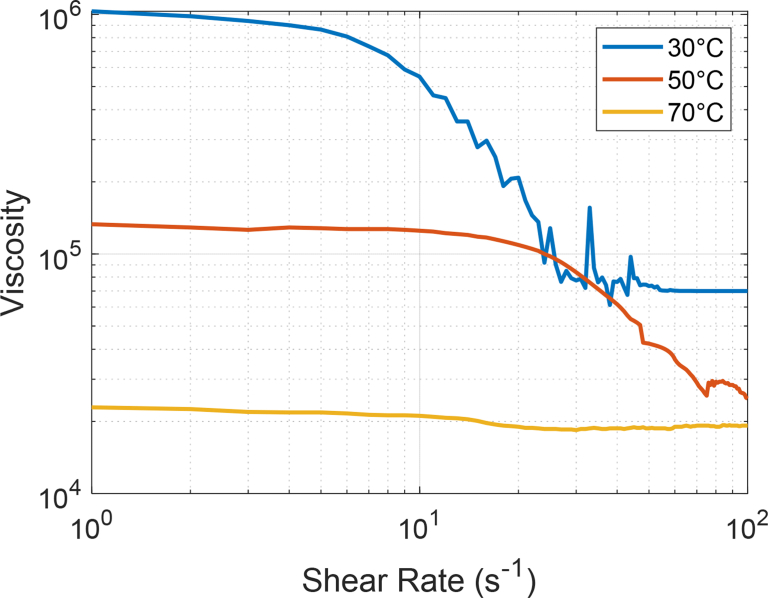
Rheological data of an extra heavy crude oil at 30 °C (303.15 K), 50 °C (323.15 K) and 70 °C (343.15 K), 1–100 s^−1^.

Magneto-rheology is an area of active research, mainly in the field of materials, recovery, and transportation of hydrocarbons, [2], [3], [4], [5]. The dataset included in this article contains experimental results showing the effect of a static magnetic field on the viscosity of several samples of an extra heavy Colombian crude oil. It is necessary to note that no magnetic material was added to the crude samples. In this section, we report information related to the variation of the viscosity due to the effect of temperature changes, sequential variation of the shear rate and exact fine-tuning of an external static magnetic field. Fig. 1 shows the simultaneous effect of the temperature and shear rate variation on the viscosity of an extra heavy crude oil sample. In this case, the temperatures were 30, 50 and 70 °C, respectively, while the shear rate changed from 1 to 100 s^−1^.

**Fig. 2:**
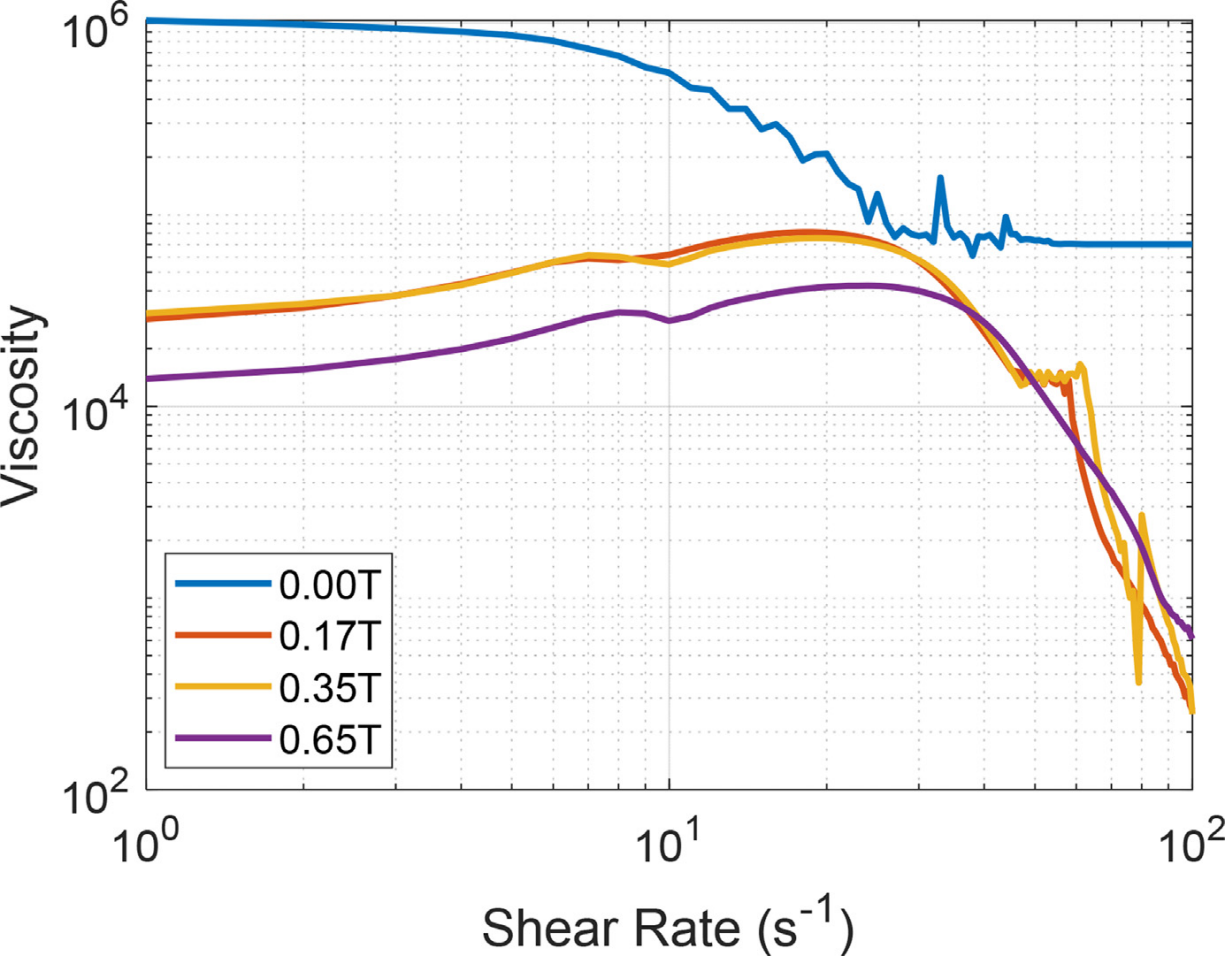
Magneto-Rheological data of an extra heavy crude oil at 30 °C (303.15 K) in the presence of a magnetic field of 0.17 T, 0.35 T and 0.65 T, respectively.

Similarly, Fig. 2 shows the effect of temperature, shear rate and the external static magnetic field on a sample viscosity, but at 30 °C, adjusting the external magnetic field to 0.17, 0.35 and 0.65 T, respectively.

**Fig. 3:**
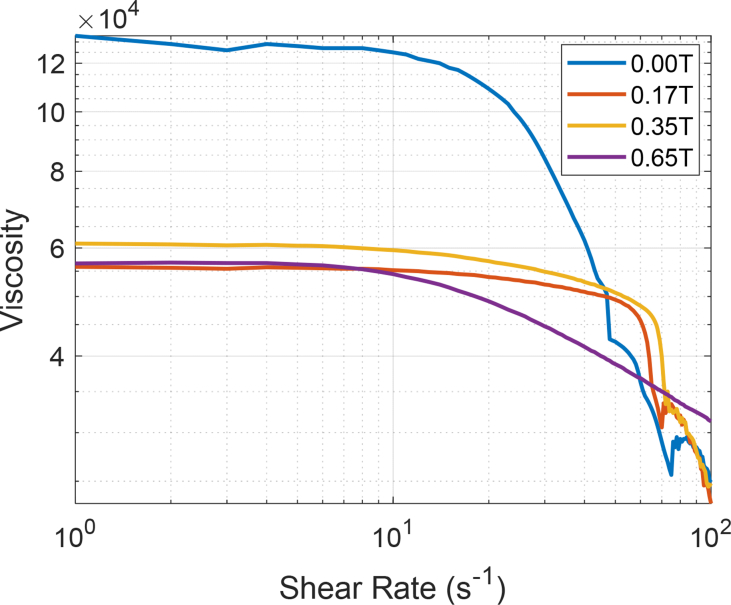
Magneto-Rheological data of an extra heavy crude oil at 50 °C (323.15 K) in the presence of a magnetic field of 0.17 T, 0.35 T and 0.65 T, respectively.

Fig. 3 includes the variation of the viscosity of a crude oil sample but, this time, at T = 50 °C. The external magnetic field values were preserved, and the shear rate changed from 1 to 100 s^−1^.

**Fig. 4:**
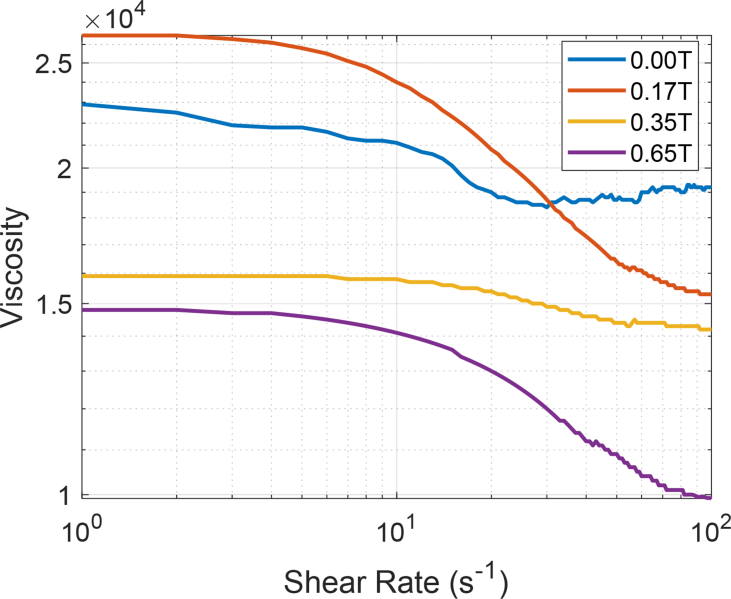
Magneto-Rheological data an extra heavy crude oil at 70 °C (343.15 K) in the presence of a magnetic field of 0.17 T, 0.35 T and 0.65 T, respectively.

Finally, Fig. 4 describes the variation of the viscosity due to the variation of the external magnetic field and the shear rate, but in this case, at T = 70 °C. Other forms of representation can be made from the raw data for all the experiments carried out. The dataset includes 12 tables (12,000 rows, 7200 measurements) and 4 figures.

## 2 Experimental design, materials, and methods

The dataset includes in detail the values of the magneto-rheological parameters which are described as follows: Shear Rate [s^−1^]; Shear Stress [Pa]; Viscosity [Pa·s]; Rotational Speed [min^−1^]; Torque [μNm] and Viscosity [Cp]. The viscosity data were acquired using an Anton Paar Rheometer MCR 302 with an online measurement of magnetic flux density. The heavy crude sample was placed in the viscometer using the cone-plate geometry. The experiments were carried out at three temperatures: 30 °C (303.15 K), 50 °C (323.15 K) and 70 °C (343.15 K). The shear rate was increased and controlled from 1 to 100 [s^−1^]. In the same way, another set of experimental data was obtained, applying a static magnetic field (also known in the technical literature as magnetic flux density or magnetic induction in Teslas [T]) of 0.17, 0.35, 0.65 T to extra heavy crude samples. The same experimental methodology was applied for all the crude oil samples. The samples were kept in an oven at room temperature at least 24 hours before the experiments. Each sample was randomly selected. No experiment duplication was allowed using the same sample. After each one of the experiments was completed, the data was automatically saved by the rheometer. From the experimental data, it was possible to generate the rheological curve of the oil crude with and without the presence of a static magnetic field. Data tables are included. Fig. 1, Fig. 2, Fig. 3, Fig. 4 compiled a series of experiments at certain temperature conditions, a range of shear rates and the presence or not of a magnetic field.
