# Supplementary material for: Magneto-rheological dataset for an extra heavy crude oil (8.5°API) in the presence of a constant magnetic field
Source: Data Brief. 2019 Apr 5;24:103902. doi: 10.1016/j.dib.2019.103902 (PMC6479066; doi:10.1016/j.dib.2019.103902)
Supplement: Multimedia component 2 [file mmc2.docx]

**Table 1.** Rheological data of an extra heavy crude oil at 303.15 K (30°C), 1-100 s^-1^

| Shear Rate | Shear Stress | Viscosity | Speed | Torque | Viscosity |
| --- | --- | --- | --- | --- | --- |
| [1/s] | [Pa] | [Pa·s] | [1/min] | [µNm] | Cp |
| 1 | 1.030 | 1.030 | 0,477 | 1.610 | 1030000 |
| 2 | 1.960 | 982 | 0,955 | 3.080 | 982000 |
| 3 | 2.820 | 939 | 1,43 | 4.410 | 939000 |
| 4 | 3.610 | 901 | 1,91 | 5.640 | 901000 |
| 5 | 4.320 | 864 | 2,39 | 6.760 | 864000 |
| 6 | 4.840 | 808 | 2,86 | 7.580 | 808000 |
| 7 | 5.150 | 735 | 3,34 | 8.060 | 735000 |
| 8 | 5.400 | 675 | 3,82 | 8.460 | 675000 |
| 9 | 5.300 | 589 | 4,3 | 8.300 | 589000 |
| 9,99 | 5.490 | 550 | 4,77 | 8.600 | 550000 |
| 11 | 5.050 | 459 | 5,25 | 7.910 | 459000 |
| 12 | 5.360 | 447 | 5,73 | 8.390 | 447000 |
| 13 | 4.650 | 357 | 6,21 | 7.270 | 357000 |
| 14 | 5.000 | 357 | 6,69 | 7.830 | 357000 |
| 15 | 4.180 | 279 | 7,16 | 6.550 | 279000 |
| 16 | 4.750 | 297 | 7,64 | 7.440 | 297000 |
| 17 | 4.320 | 254 | 8,12 | 6.760 | 254000 |
| 18 | 3.460 | 192 | 8,59 | 5.420 | 192000 |
| 19 | 3.920 | 206 | 9,07 | 6.140 | 206000 |
| 20 | 4.160 | 208 | 9,55 | 6.520 | 208000 |
| 21 | 3.500 | 167 | 10 | 5.480 | 167000 |
| 22 | 3.190 | 145 | 10,5 | 4.990 | 145000 |
| 23 | 3.130 | 136 | 11 | 4.890 | 136000 |
| 24 | 2.210 | 92 | 11,5 | 3.460 | 92000 |
| 25 | 3.210 | 128 | 11,9 | 5.020 | 128000 |
| 26 | 2.350 | 90,3 | 12,4 | 3.680 | 90300 |
| 27 | 2.070 | 76,5 | 12,9 | 3.230 | 76500 |
| 28 | 2.380 | 85 | 13,4 | 3.730 | 85000 |
| 29 | 1.430 | 79,2 | 13,8 | 2.240 | 79200 |
| 30 | 1.720 | 77,5 | 14,3 | 2.700 | 77500 |
| 31 | 1.200 | 78,7 | 14,8 | 1.880 | 78700 |
| 32 | 1.670 | 72,1 | 15,3 | 2.610 | 72100 |
| 33 | 5.130 | 156 | 15,7 | 8.030 | 156000 |
| 34 | 2.970 | 87,4 | 16,2 | 4.650 | 87400 |
| 35 | 914 | 76,1 | 16,7 | 1.430 | 76100 |
| 36 | 711 | 79,8 | 17,2 | 1.110 | 79800 |
| 37 | 1.630 | 74,1 | 17,7 | 2.560 | 74100 |
| 38 | 2.330 | 61,2 | 18,1 | 3.640 | 61200 |
| 39 | 657 | 76,8 | 18,6 | 1.030 | 76800 |
| 40 | 1.060 | 76,4 | 19,1 | 1.650 | 76400 |
| 41 | 761 | 78,6 | 19,6 | 1.190 | 78600 |
| 42 | 1.790 | 72,6 | 20 | 2.800 | 72600 |
| 43 | 2.900 | 67,5 | 20,5 | 4.540 | 67500 |
| 44 | 4.280 | 97,3 | 21 | 6.700 | 97300 |
| 45 | 864 | 79,2 | 21,5 | 1.350 | 79200 |
| 46 | 422 | 79,17 | 22 | 661 | 79170 |
| 47 | 658 | 74 | 22,4 | 1.030 | 74000 |
| 48 | 214 | 74,46 | 22,9 | 335 | 74460 |
| 49 | 217 | 74,42 | 23,4 | 339 | 74420 |
| 50 | 2.670 | 73,4 | 23,9 | 4.180 | 73400 |
| 51 | 697 | 73,7 | 24,4 | 1.090 | 73700 |
| 52 | 123 | 72,36 | 24,8 | 192 | 72360 |
| 53 | 167 | 73,16 | 25,3 | 262 | 73160 |
| 54 | 37,2 | 70,69 | 25,8 | 58,3 | 70690 |
| 55 | 18,8 | 70,343 | 26,3 | 29,5 | 70343 |
| 56 | 12,4 | 70,222 | 26,7 | 19,4 | 70222 |
| 57 | 9,38 | 70,165 | 27,2 | 14,7 | 70165 |
| 58 | 40,7 | 70,702 | 27,7 | 63,8 | 70702 |
| 59 | 26,1 | 70,443 | 28,2 | 40,9 | 70443 |
| 60 | 14,2 | 70,237 | 28,6 | 22,3 | 70237 |
| 61 | 8,76 | 70,144 | 29,1 | 13,7 | 70144 |
| 62 | 6,81 | 70,11 | 29,6 | 10,7 | 70110 |
| 63 | 5,7 | 70,0904 | 30,1 | 8,92 | 70090,4 |
| 64 | 4,96 | 70,0775 | 30,6 | 7,77 | 70077,5 |
| 65 | 4,47 | 70,0687 | 31 | 6,99 | 70068,7 |
| 66 | 4,06 | 70,0615 | 31,5 | 6,36 | 70061,5 |
| 67 | 3,76 | 70,0561 | 32 | 5,88 | 70056,1 |
| 68 | 3,47 | 70,051 | 32,5 | 5,43 | 70051 |
| 69 | 3,23 | 70,0469 | 32,9 | 5,06 | 70046,9 |
| 70 | 3 | 70,0429 | 33,4 | 4,7 | 70042,9 |
| 71 | 2,81 | 70,0396 | 33,9 | 4,4 | 70039,6 |
| 72 | 2,64 | 70,0367 | 34,4 | 4,14 | 70036,7 |
| 73 | 2,49 | 70,0341 | 34,8 | 3,9 | 70034,1 |
| 74 | 2,32 | 70,0314 | 35,3 | 3,63 | 70031,4 |
| 75 | 2,2 | 70,0293 | 35,8 | 3,44 | 70029,3 |
| 76 | 2,08 | 70,0273 | 36,3 | 3,25 | 70027,3 |
| 77 | 1,94 | 70,0253 | 36,8 | 3,04 | 70025,3 |
| 78 | 1,8 | 70,0231 | 37,2 | 2,82 | 70023,1 |
| 79 | 1,63 | 70,0207 | 37,7 | 2,56 | 70020,7 |
| 80 | 1,52 | 70,019 | 38,2 | 2,38 | 70019 |
| 81 | 1,4 | 70,0173 | 38,7 | 2,19 | 70017,3 |
| 82 | 1,28 | 70,0157 | 39,1 | 2,01 | 70015,7 |
| 83 | 1,16 | 70,0139 | 39,6 | 1,81 | 70013,9 |
| 84 | 0,931 | 70,0111 | 40,1 | 1,46 | 70011,1 |
| 85 | 1,1 | 70,013 | 40,6 | 1,72 | 70013 |
| 86 | 1,24 | 70,0144 | 41,1 | 1,93 | 70014,4 |
| 87 | 0,899 | 70,0103 | 41,5 | 1,41 | 70010,3 |
| 88 | 0,796 | 70,00905 | 42 | 1,25 | 70009,05 |
| 89 | 0,63 | 70,00708 | 42,5 | 0,987 | 70007,08 |
| 90 | 0,566 | 70,00629 | 43 | 0,887 | 70006,29 |
| 91 | 0,526 | 70,00578 | 43,4 | 0,824 | 70005,78 |
| 92 | 0,414 | 70,0045 | 43,9 | 0,648 | 70004,5 |
| 93 | 0,423 | 70,00455 | 44,4 | 0,662 | 70004,55 |
| 94 | 0,399 | 70,00424 | 44,9 | 0,624 | 70004,24 |
| 95 | 0,385 | 70,00405 | 45,3 | 0,602 | 70004,05 |
| 96 | 0,344 | 70,00359 | 45,8 | 0,539 | 70003,59 |
| 97 | 0,377 | 70,00389 | 46,3 | 0,591 | 70003,89 |
| 98 | 0,38 | 70,00387 | 46,8 | 0,594 | 70003,87 |
| 99 | 0,42 | 70,00424 | 47,3 | 0,657 | 70004,24 |
| 100 | 0,403 | 70,00403 | 47,7 | 0,631 | 70004,03 |

**Table 2.** Rheological data of an extra heavy crude oil at 303.15 K (30°C), 1-100 s^-1^, 0.17 T

| Shear Rate | Shear Stress | Viscosity | Speed | Torque | Viscosity |
| --- | --- | --- | --- | --- | --- |
| [1/s] | [Pa] | [Pa·s] | [1/min] | [µNm] | Cp |
| 1 | 0,892 | 28,544 | 0,477 | 1,4 | 28544 |
| 2 | 2,05 | 32,64 | 0,955 | 3,21 | 32640 |
| 3 | 3,53 | 37,76 | 1,43 | 5,53 | 37760 |
| 4 | 5,46 | 43,52 | 1,91 | 8,54 | 43520 |
| 5 | 7,82 | 49,92 | 2,39 | 12,2 | 49920 |
| 6 | 10,5 | 56,32 | 2,86 | 16,5 | 56320 |
| 7 | 12,9 | 59,2 | 3,34 | 20,2 | 59200 |
| 8 | 14,5 | 57,92 | 3,82 | 22,7 | 57920 |
| 9 | 16,7 | 59,52 | 4,3 | 26,2 | 59520 |
| 10 | 19,3 | 61,76 | 4,77 | 30,3 | 61760 |
| 11 | 22,8 | 66,24 | 5,25 | 35,7 | 66240 |
| 12 | 26,4 | 70,4 | 5,73 | 41,4 | 70400 |
| 13 | 29,8 | 73,28 | 6,21 | 46,7 | 73280 |
| 14 | 33,2 | 75,84 | 6,68 | 51,9 | 75840 |
| 15 | 36,7 | 78,4 | 7,16 | 57,4 | 78400 |
| 16 | 39,9 | 79,68 | 7,64 | 62,5 | 79680 |
| 17 | 42,8 | 80,64 | 8,12 | 67,1 | 80640 |
| 18 | 45,6 | 80,96 | 8,59 | 71,3 | 80960 |
| 19 | 48 | 80,96 | 9,07 | 75,2 | 80960 |
| 20 | 50,3 | 80,32 | 9,55 | 78,7 | 80320 |
| 21 | 52,2 | 79,68 | 10 | 81,7 | 79680 |
| 22 | 53,8 | 78,08 | 10,5 | 84,2 | 78080 |
| 23 | 55,1 | 76,48 | 11 | 86,2 | 76480 |
| 24 | 55,9 | 74,56 | 11,5 | 87,5 | 74560 |
| 25 | 56,5 | 72,32 | 11,9 | 88,4 | 72320 |
| 26 | 56,6 | 69,76 | 12,4 | 88,7 | 69760 |
| 27 | 56,4 | 66,88 | 12,9 | 88,3 | 66880 |
| 28 | 55,8 | 63,68 | 13,4 | 87,4 | 63680 |
| 29 | 54,9 | 60,48 | 13,8 | 85,9 | 60480 |
| 30 | 53,5 | 56,96 | 14,3 | 83,7 | 56960 |
| 31 | 51,8 | 53,44 | 14,8 | 81,1 | 53440 |
| 32 | 49,7 | 49,6 | 15,3 | 77,8 | 49600 |
| 33 | 47,3 | 45,76 | 15,8 | 74,1 | 45760 |
| 34 | 44,8 | 42,24 | 16,2 | 70,2 | 42240 |
| 35 | 42,3 | 38,72 | 16,7 | 66,2 | 38720 |
| 36 | 39,7 | 35,2 | 17,2 | 62,2 | 35200 |
| 37 | 37,2 | 32,32 | 17,7 | 58,3 | 32320 |
| 38 | 34,8 | 29,312 | 18,1 | 54,5 | 29312 |
| 39 | 32,6 | 26,72 | 18,6 | 51 | 26720 |
| 40 | 30,4 | 24,352 | 19,1 | 47,6 | 24352 |
| 41 | 28,5 | 22,208 | 19,6 | 44,6 | 22208 |
| 42 | 26,6 | 20,288 | 20 | 41,7 | 20288 |
| 43 | 24,9 | 18,56 | 20,5 | 39 | 18560 |
| 44 | 23,3 | 16,992 | 21 | 36,5 | 16992 |
| 45 | 21,8 | 15,488 | 21,5 | 34,1 | 15488 |
| 46 | 40,3 | 15,232 | 22 | 63 | 15232 |
| 47 | 66,1 | 14,944 | 22,4 | 104 | 14944 |
| 48 | 61,2 | 13,664 | 22,9 | 95,8 | 13664 |
| 49 | 49,5 | 13,44 | 23,4 | 77,5 | 13440 |
| 50 | 36,1 | 13,504 | 23,9 | 56,5 | 13504 |
| 51 | 44 | 14,816 | 24,3 | 68,9 | 14816 |
| 52 | 55 | 12,992 | 24,8 | 86,2 | 12992 |
| 53 | 40,2 | 14,688 | 25,3 | 63 | 14688 |
| 54 | 49,6 | 13,376 | 25,8 | 77,6 | 13376 |
| 55 | 38,9 | 13,056 | 26,3 | 61 | 13056 |
| 56 | 26,1 | 14,944 | 26,7 | 40,9 | 14944 |
| 57 | 37,8 | 11,648 | 27,2 | 59,2 | 11648 |
| 58 | 24,8 | 13,696 | 27,7 | 38,8 | 13696 |
| 59 | 15,7 | 8,544 | 28,2 | 24,6 | 8544 |
| 60 | 13 | 6,912 | 28,6 | 20,3 | 6912 |
| 61 | 10,1 | 5,28 | 29,1 | 15,7 | 5280 |
| 62 | 8,47 | 4,384 | 29,6 | 13,3 | 4384 |
| 63 | 7,31 | 3,712 | 30,1 | 11,4 | 3712 |
| 64 | 6,38 | 3,1872 | 30,6 | 9,98 | 3187,2 |
| 65 | 5,66 | 2,784 | 31 | 8,85 | 2784 |
| 66 | 5,05 | 2,4512 | 31,5 | 7,91 | 2451,2 |
| 67 | 4,55 | 2,1728 | 32 | 7,12 | 2172,8 |
| 68 | 4,21 | 1,984 | 32,5 | 6,6 | 1984 |
| 69 | 3,98 | 1,8464 | 32,9 | 6,24 | 1846,4 |
| 70 | 3,75 | 1,712 | 33,4 | 5,87 | 1712 |
| 71 | 3,43 | 1,5456 | 33,9 | 5,36 | 1545,6 |
| 72 | 3,36 | 1,4912 | 34,4 | 5,25 | 1491,2 |
| 73 | 3,17 | 1,392 | 34,8 | 4,97 | 1392 |
| 74 | 3,05 | 1,3184 | 35,3 | 4,77 | 1318,4 |
| 75 | 2,91 | 1,2416 | 35,8 | 4,55 | 1241,6 |
| 76 | 2,81 | 1,184 | 36,3 | 4,4 | 1184 |
| 77 | 2,65 | 1,1008 | 36,8 | 4,14 | 1100,8 |
| 78 | 2,54 | 1,0432 | 37,2 | 3,98 | 1043,2 |
| 79 | 2,41 | 0,9792 | 37,7 | 3,78 | 979,2 |
| 80 | 2,29 | 0,9152 | 38,2 | 3,58 | 915,2 |
| 81 | 2,21 | 0,8736 | 38,7 | 3,46 | 873,6 |
| 82 | 2,09 | 0,816 | 39,1 | 3,28 | 816 |
| 83 | 1,99 | 0,768 | 39,6 | 3,12 | 768 |
| 84 | 1,83 | 0,6976 | 40,1 | 2,86 | 697,6 |
| 85 | 1,77 | 0,6656 | 40,6 | 2,77 | 665,6 |
| 86 | 1,69 | 0,6272 | 41,1 | 2,64 | 627,2 |
| 87 | 1,63 | 0,5984 | 41,5 | 2,55 | 598,4 |
| 88 | 1,51 | 0,5504 | 42 | 2,36 | 550,4 |
| 89 | 1,4 | 0,5056 | 42,5 | 2,2 | 505,6 |
| 90 | 1,39 | 0,496 | 43 | 2,18 | 496 |
| 91 | 1,27 | 0,448 | 43,4 | 1,99 | 448 |
| 92 | 1,29 | 0,4512 | 43,9 | 2,03 | 451,2 |
| 93 | 1,16 | 0,4 | 44,4 | 1,82 | 400 |
| 94 | 1,11 | 0,3808 | 44,9 | 1,75 | 380,8 |
| 95 | 1,08 | 0,3648 | 45,3 | 1,69 | 364,8 |
| 96 | 1,02 | 0,3392 | 45,8 | 1,59 | 339,2 |
| 97 | 0,922 | 0,304 | 46,3 | 1,44 | 304 |
| 98 | 1 | 0,3264 | 46,8 | 1,56 | 326,4 |
| 99 | 0,857 | 0,27712 | 47,3 | 1,34 | 277,12 |
| 100 | 0,812 | 0,25984 | 47,7 | 1,27 | 259,84 |

**Table 3.** Rheological data of an extra heavy crude oil at 303.15 K (30°C), 1-100 s^-1^, 0.35 T

| Shear Rate | Shear Stress | Viscosity | Speed | Torque | Viscosity |
| --- | --- | --- | --- | --- | --- |
| [1/s] | [Pa] | [Pa·s] | [1/min] | [µNm] | Cp |
| 1 | 0,953 | 30,528 | 0,477 | 1,49 | 30528 |
| 2 | 2,13 | 34,24 | 0,954 | 3,34 | 34240 |
| 3 | 3,54 | 37,76 | 1,43 | 5,55 | 37760 |
| 4 | 5,36 | 42,88 | 1,91 | 8,39 | 42880 |
| 5 | 7,75 | 49,6 | 2,39 | 12,1 | 49600 |
| 6 | 10,6 | 56,64 | 2,86 | 16,7 | 56640 |
| 7 | 13,5 | 61,44 | 3,34 | 21,1 | 61440 |
| 8 | 15,1 | 60,48 | 3,82 | 23,7 | 60480 |
| 9 | 16,1 | 56,96 | 4,3 | 25,1 | 56960 |
| 10 | 17,2 | 55,04 | 4,77 | 27 | 55040 |
| 11 | 20,4 | 59,52 | 5,25 | 31,9 | 59520 |
| 12 | 24,3 | 64,96 | 5,73 | 38 | 64960 |
| 13 | 27,6 | 67,84 | 6,21 | 43,2 | 67840 |
| 14 | 30,8 | 70,4 | 6,68 | 48,2 | 70400 |
| 15 | 33,8 | 72 | 7,16 | 52,9 | 72000 |
| 16 | 36,9 | 73,6 | 7,64 | 57,7 | 73600 |
| 17 | 39,7 | 74,56 | 8,11 | 62,1 | 74560 |
| 18 | 42,3 | 75,2 | 8,59 | 66,3 | 75200 |
| 19 | 44,8 | 75,52 | 9,07 | 70,2 | 75520 |
| 20 | 47 | 75,2 | 9,55 | 73,7 | 75200 |
| 21 | 49,1 | 74,88 | 10 | 76,8 | 74880 |
| 22 | 50,8 | 73,92 | 10,5 | 79,5 | 73920 |
| 23 | 52,3 | 72,64 | 11 | 81,9 | 72640 |
| 24 | 53,5 | 71,36 | 11,5 | 83,8 | 71360 |
| 25 | 54,4 | 69,76 | 11,9 | 85,2 | 69760 |
| 26 | 55 | 67,84 | 12,4 | 86,1 | 67840 |
| 27 | 55,2 | 65,6 | 12,9 | 86,5 | 65600 |
| 28 | 55,2 | 63,04 | 13,4 | 86,5 | 63040 |
| 29 | 54,9 | 60,48 | 13,8 | 86 | 60480 |
| 30 | 54,2 | 57,92 | 14,3 | 84,8 | 57920 |
| 31 | 53,2 | 55,04 | 14,8 | 83,3 | 55040 |
| 32 | 51,7 | 51,84 | 15,3 | 80,9 | 51840 |
| 33 | 50 | 48,32 | 15,8 | 78,2 | 48320 |
| 34 | 47,9 | 45,12 | 16,2 | 74,9 | 45120 |
| 35 | 45,5 | 41,6 | 16,7 | 71,3 | 41600 |
| 36 | 43 | 38,08 | 17,2 | 67,3 | 38080 |
| 37 | 40,4 | 34,88 | 17,7 | 63,2 | 34880 |
| 38 | 38,1 | 32 | 18,1 | 59,6 | 32000 |
| 39 | 35,6 | 29,216 | 18,6 | 55,8 | 29216 |
| 40 | 33 | 26,432 | 19,1 | 51,7 | 26432 |
| 41 | 30,5 | 23,776 | 19,6 | 47,7 | 23776 |
| 42 | 28,1 | 21,376 | 20 | 43,9 | 21376 |
| 43 | 25,9 | 19,296 | 20,5 | 40,6 | 19296 |
| 44 | 23,9 | 17,344 | 21 | 37,4 | 17344 |
| 45 | 22,1 | 15,712 | 21,5 | 34,6 | 15712 |
| 46 | 20,5 | 14,24 | 22 | 32,1 | 14240 |
| 47 | 18,9 | 12,832 | 22,4 | 29,5 | 12832 |
| 48 | 67,6 | 13,12 | 22,9 | 106 | 13120 |
| 49 | 52,4 | 15,04 | 23,4 | 82,1 | 15040 |
| 50 | 61,5 | 13,76 | 23,9 | 96,2 | 13760 |
| 51 | 39,3 | 15,04 | 24,3 | 61,5 | 15040 |
| 52 | 52,6 | 13,12 | 24,8 | 82,3 | 13120 |
| 53 | 56,8 | 15,04 | 25,3 | 88,9 | 15040 |
| 54 | 39,9 | 14,016 | 25,8 | 62,4 | 14016 |
| 55 | 51,2 | 13,792 | 26,3 | 80,1 | 13792 |
| 56 | 31,5 | 14,784 | 26,7 | 49,3 | 14784 |
| 57 | 41,2 | 13,536 | 27,2 | 64,5 | 13536 |
| 58 | 44,2 | 14,752 | 27,7 | 69,1 | 14752 |
| 59 | 27,3 | 14,816 | 28,2 | 42,7 | 14816 |
| 60 | 50,8 | 14,272 | 28,6 | 79,5 | 14272 |
| 61 | 31,5 | 16,544 | 29,1 | 49,4 | 16544 |
| 62 | 30 | 15,456 | 29,6 | 46,9 | 15456 |
| 63 | 22,5 | 11,424 | 30,1 | 35,2 | 11424 |
| 64 | 18,6 | 9,28 | 30,5 | 29,1 | 9280 |
| 65 | 13,1 | 6,432 | 31 | 20,5 | 6432 |
| 66 | 10,1 | 4,896 | 31,5 | 15,8 | 4896 |
| 67 | 8,42 | 4,032 | 32 | 13,2 | 4032 |
| 68 | 7,27 | 3,424 | 32,5 | 11,4 | 3424 |
| 69 | 6,42 | 2,9792 | 32,9 | 10,1 | 2979,2 |
| 70 | 5,84 | 2,6688 | 33,4 | 9,14 | 2668,8 |
| 71 | 5,23 | 2,3584 | 33,9 | 8,19 | 2358,4 |
| 72 | 4,78 | 2,1216 | 34,4 | 7,48 | 2121,6 |
| 73 | 40,1 | 1,7568 | 34,8 | 62,7 | 1756,8 |
| 74 | 44,8 | 1,936 | 35,3 | 70,1 | 1936 |
| 75 | 28 | 1,1936 | 35,8 | 43,8 | 1193,6 |
| 76 | 23,9 | 1,0048 | 36,3 | 37,4 | 1004,8 |
| 77 | 26,2 | 1,088 | 36,8 | 40,9 | 1088 |
| 78 | 13,6 | 0,56 | 37,2 | 21,4 | 560 |
| 79 | 8,96 | 0,3616 | 37,7 | 14 | 361,6 |
| 80 | 6,78 | 2,7136 | 38,2 | 10,6 | 2713,6 |
| 81 | 5,54 | 2,1888 | 38,7 | 8,67 | 2188,8 |
| 82 | 4,7 | 1,8336 | 39,1 | 7,35 | 1833,6 |
| 83 | 4,12 | 1,5872 | 39,6 | 6,45 | 1587,2 |
| 84 | 3,65 | 1,392 | 40,1 | 5,72 | 1392 |
| 85 | 3,25 | 1,2224 | 40,6 | 5,09 | 1222,4 |
| 86 | 2,95 | 1,0976 | 41 | 4,62 | 1097,6 |
| 87 | 2,66 | 0,9792 | 41,5 | 4,17 | 979,2 |
| 88 | 2,46 | 0,8928 | 42 | 3,84 | 892,8 |
| 89 | 2,24 | 0,8032 | 42,5 | 3,5 | 803,2 |
| 90 | 2,08 | 0,7392 | 43 | 3,25 | 739,2 |
| 91 | 1,99 | 0,6976 | 43,4 | 3,11 | 697,6 |
| 92 | 1,73 | 0,6016 | 43,9 | 2,71 | 601,6 |
| 93 | 1,62 | 0,5568 | 44,4 | 2,53 | 556,8 |
| 94 | 1,42 | 0,4832 | 44,9 | 2,22 | 483,2 |
| 95 | 1,42 | 0,4768 | 45,3 | 2,22 | 476,8 |
| 96 | 1,22 | 0,4064 | 45,8 | 1,91 | 406,4 |
| 97 | 1,19 | 0,3936 | 46,3 | 1,86 | 393,6 |
| 98 | 1,16 | 0,3776 | 46,8 | 1,82 | 377,6 |
| 99 | 1,07 | 0,3456 | 47,3 | 1,67 | 345,6 |
| 100 | 0,775 | 0,248 | 47,7 | 1,21 | 248 |

**Table 4.** Rheological data of a heavy crude oil at 303.15 K (30°C), 1-100 s^-1^, 0.65 T

| Shear Rate | Shear Stress | Viscosity | Speed | Torque | Viscosity |
| --- | --- | --- | --- | --- | --- |
| [1/s] | [Pa] | [Pa·s] | [1/min] | [µNm] | Cp |
| 1 | 0,435 | 13,92 | 0,477 | 0,681 | 13920 |
| 2 | 0,971 | 15,552 | 0,955 | 1,52 | 15552 |
| 3 | 1,65 | 17,6 | 1,43 | 2,58 | 17600 |
| 4 | 2,48 | 19,84 | 1,91 | 3,88 | 19840 |
| 5 | 3,52 | 22,528 | 2,39 | 5,51 | 22528 |
| 6 | 4,82 | 25,728 | 2,86 | 7,55 | 25728 |
| 7 | 6,32 | 28,896 | 3,34 | 9,9 | 28896 |
| 8 | 7,74 | 30,944 | 3,82 | 12,1 | 30944 |
| 9 | 8,56 | 30,464 | 4,3 | 13,4 | 30464 |
| 10 | 8,72 | 27,904 | 4,77 | 13,6 | 27904 |
| 11 | 10,1 | 29,472 | 5,25 | 15,9 | 29472 |
| 12 | 12,3 | 32,64 | 5,73 | 19,2 | 32640 |
| 13 | 14,1 | 34,88 | 6,2 | 22,1 | 34880 |
| 14 | 16 | 36,48 | 6,68 | 25 | 36480 |
| 15 | 17,7 | 37,76 | 7,16 | 27,8 | 37760 |
| 16 | 19,5 | 39,04 | 7,64 | 30,5 | 39040 |
| 17 | 21,3 | 40 | 8,11 | 33,3 | 40000 |
| 18 | 23 | 40,96 | 8,59 | 35,9 | 40960 |
| 19 | 24,6 | 41,6 | 9,07 | 38,6 | 41600 |
| 20 | 26,3 | 41,92 | 9,55 | 41,1 | 41920 |
| 21 | 27,8 | 42,24 | 10 | 43,5 | 42240 |
| 22 | 29,3 | 42,56 | 10,5 | 45,9 | 42560 |
| 23 | 30,7 | 42,56 | 11 | 48,1 | 42560 |
| 24 | 32 | 42,56 | 11,5 | 50,1 | 42560 |
| 25 | 33,3 | 42,56 | 11,9 | 52,1 | 42560 |
| 26 | 34,4 | 42,24 | 12,4 | 53,8 | 42240 |
| 27 | 35,3 | 41,92 | 12,9 | 55,3 | 41920 |
| 28 | 36,1 | 41,28 | 13,4 | 56,6 | 41280 |
| 29 | 36,8 | 40,64 | 13,8 | 57,7 | 40640 |
| 30 | 37,4 | 40 | 14,3 | 58,5 | 40000 |
| 31 | 37,8 | 39,04 | 14,8 | 59,2 | 39040 |
| 32 | 38,1 | 38,08 | 15,3 | 59,6 | 38080 |
| 33 | 38,2 | 37,12 | 15,7 | 59,8 | 37120 |
| 34 | 38,1 | 35,84 | 16,2 | 59,7 | 35840 |
| 35 | 37,9 | 34,56 | 16,7 | 59,4 | 34560 |
| 36 | 37,6 | 33,28 | 17,2 | 58,8 | 33280 |
| 37 | 37 | 32 | 17,7 | 58 | 32000 |
| 38 | 36,3 | 30,592 | 18,1 | 56,8 | 30592 |
| 39 | 35,4 | 29,056 | 18,6 | 55,4 | 29056 |
| 40 | 34,3 | 27,456 | 19,1 | 53,7 | 27456 |
| 41 | 33,1 | 25,824 | 19,6 | 51,8 | 25824 |
| 42 | 31,7 | 24,192 | 20 | 49,7 | 24192 |
| 43 | 30,3 | 22,56 | 20,5 | 47,5 | 22560 |
| 44 | 28,8 | 20,928 | 21 | 45,1 | 20928 |
| 45 | 27,3 | 19,424 | 21,5 | 42,7 | 19424 |
| 46 | 25,8 | 17,952 | 22 | 40,4 | 17952 |
| 47 | 24,3 | 16,576 | 22,4 | 38,1 | 16576 |
| 48 | 23 | 15,328 | 22,9 | 36 | 15328 |
| 49 | 21,7 | 14,176 | 23,4 | 34 | 14176 |
| 50 | 20,4 | 13,056 | 23,9 | 32 | 13056 |
| 51 | 19,3 | 12,128 | 24,3 | 30,2 | 12128 |
| 52 | 18,3 | 11,232 | 24,8 | 28,6 | 11232 |
| 53 | 17,3 | 10,432 | 25,3 | 27,1 | 10432 |
| 54 | 16,4 | 9,696 | 25,8 | 25,6 | 9696 |
| 55 | 15,6 | 9,056 | 26,2 | 24,4 | 9056 |
| 56 | 14,7 | 8,416 | 26,7 | 23,1 | 8416 |
| 57 | 14 | 7,872 | 27,2 | 21,9 | 7872 |
| 58 | 13,4 | 7,36 | 27,7 | 20,9 | 7360 |
| 59 | 12,7 | 6,912 | 28,2 | 19,9 | 6912 |
| 60 | 12,1 | 6,432 | 28,6 | 18,9 | 6432 |
| 61 | 11,5 | 6,016 | 29,1 | 18 | 6016 |
| 62 | 10,9 | 5,632 | 29,6 | 17,1 | 5632 |
| 63 | 10,4 | 5,312 | 30,1 | 16,3 | 5312 |
| 64 | 9,99 | 4,992 | 30,5 | 15,6 | 4992 |
| 65 | 9,59 | 4,736 | 31 | 15 | 4736 |
| 66 | 9,22 | 4,48 | 31,5 | 14,4 | 4480 |
| 67 | 8,85 | 4,224 | 32 | 13,9 | 4224 |
| 68 | 8,43 | 3,968 | 32,5 | 13,2 | 3968 |
| 69 | 8,06 | 3,744 | 32,9 | 12,6 | 3744 |
| 70 | 7,81 | 3,584 | 33,4 | 12,2 | 3584 |
| 71 | 7,43 | 3,36 | 33,9 | 11,6 | 3360 |
| 72 | 7,11 | 3,1584 | 34,4 | 11,1 | 3158,4 |
| 73 | 6,81 | 2,9856 | 34,8 | 10,7 | 2985,6 |
| 74 | 6,48 | 2,8032 | 35,3 | 10,2 | 2803,2 |
| 75 | 6,18 | 2,6368 | 35,8 | 9,67 | 2636,8 |
| 76 | 5,88 | 2,4768 | 36,3 | 9,21 | 2476,8 |
| 77 | 5,5 | 2,2848 | 36,7 | 8,61 | 2284,8 |
| 78 | 5,21 | 2,1376 | 37,2 | 8,15 | 2137,6 |
| 79 | 4,92 | 1,9936 | 37,7 | 7,71 | 1993,6 |
| 80 | 4,62 | 1,8464 | 38,2 | 7,23 | 1846,4 |
| 81 | 4,29 | 1,6928 | 38,7 | 6,71 | 1692,8 |
| 82 | 3,95 | 1,5424 | 39,1 | 6,19 | 1542,4 |
| 83 | 3,62 | 1,3984 | 39,6 | 5,67 | 1398,4 |
| 84 | 3,39 | 1,2896 | 40,1 | 5,31 | 1289,6 |
| 85 | 3,15 | 1,1872 | 40,6 | 4,93 | 1187,2 |
| 86 | 2,94 | 1,0944 | 41 | 4,6 | 1094,4 |
| 87 | 2,76 | 1,0176 | 41,5 | 4,33 | 1017,6 |
| 88 | 2,62 | 0,9536 | 42 | 4,11 | 953,6 |
| 89 | 2,54 | 0,9152 | 42,5 | 3,98 | 915,2 |
| 90 | 2,5 | 0,8896 | 42,9 | 3,91 | 889,6 |
| 91 | 2,38 | 0,8352 | 43,4 | 3,72 | 835,2 |
| 92 | 2,32 | 0,8096 | 43,9 | 3,64 | 809,6 |
| 93 | 2,32 | 0,8 | 44,4 | 3,64 | 800 |
| 94 | 2,21 | 0,752 | 44,9 | 3,46 | 752 |
| 95 | 2,23 | 0,7488 | 45,3 | 3,49 | 748,8 |
| 96 | 2,15 | 0,7168 | 45,8 | 3,36 | 716,8 |
| 97 | 2,1 | 0,6912 | 46,3 | 3,29 | 691,2 |
| 98 | 2,16 | 0,704 | 46,8 | 3,37 | 704 |
| 99 | 2,02 | 0,6528 | 47,2 | 3,16 | 652,8 |
| 100 | 1,91 | 0,6112 | 47,7 | 2,99 | 611,2 |

**Table 5.** Rheological data of an extra heavy crude oil at 323.15 K (50°C), 1-100 s^-1^

| Shear Rate | Shear Stress | Viscosity | Speed | Torque | Viscosity |
| --- | --- | --- | --- | --- | --- |
| [1/s] | [Pa] | [Pa·s] | [1/min] | [µNm] | Cp |
| 1 | 133 | 133 | 0,478 | 208 | 133000 |
| 2 | 258 | 129 | 0,955 | 404 | 129000 |
| 3 | 379 | 126 | 1,43 | 593 | 126000 |
| 4 | 516 | 129 | 1,91 | 808 | 129000 |
| 5 | 639 | 128 | 2,39 | 1.000 | 128000 |
| 6 | 765 | 127 | 2,87 | 1.200 | 127000 |
| 7 | 890 | 127 | 3,34 | 1.390 | 127000 |
| 8 | 1.010 | 127 | 3,82 | 1.580 | 127000 |
| 9 | 1.130 | 126 | 4,3 | 1.770 | 126000 |
| 10 | 1.250 | 125 | 4,78 | 1.950 | 125000 |
| 11 | 1.360 | 124 | 5,25 | 2.130 | 124000 |
| 12 | 1.470 | 122 | 5,73 | 2.300 | 122000 |
| 13 | 1.570 | 121 | 6,21 | 2.460 | 121000 |
| 14 | 1.670 | 120 | 6,69 | 2.620 | 120000 |
| 15 | 1.770 | 118 | 7,16 | 2.770 | 118000 |
| 16 | 1.860 | 117 | 7,64 | 2.920 | 117000 |
| 17 | 1.950 | 115 | 8,12 | 3.060 | 115000 |
| 18 | 2.040 | 113 | 8,6 | 3.190 | 113000 |
| 19 | 2.110 | 111 | 9,07 | 3.310 | 111000 |
| 20 | 2.180 | 109 | 9,55 | 3.420 | 109000 |
| 21 | 2.250 | 107 | 10 | 3.520 | 107000 |
| 22 | 2.310 | 105 | 10,5 | 3.610 | 105000 |
| 23 | 2.360 | 103 | 11 | 3.690 | 103000 |
| 24 | 2.400 | 100 | 11,5 | 3.760 | 100000 |
| 25 | 2.440 | 97,7 | 11,9 | 3.820 | 97700 |
| 26 | 2.470 | 95,1 | 12,4 | 3.870 | 95100 |
| 27 | 2.490 | 92,4 | 12,9 | 3.900 | 92400 |
| 28 | 2.510 | 89,5 | 13,4 | 3.930 | 89500 |
| 29 | 2.510 | 86,7 | 13,9 | 3.940 | 86700 |
| 30 | 2.520 | 83,9 | 14,3 | 3.940 | 83900 |
| 31 | 2.510 | 81,1 | 14,8 | 3.940 | 81100 |
| 32 | 2.510 | 78,5 | 15,3 | 3.930 | 78500 |
| 33 | 2.510 | 76 | 15,8 | 3.930 | 76000 |
| 34 | 2.500 | 73,6 | 16,2 | 3.920 | 73600 |
| 35 | 2.500 | 71,4 | 16,7 | 3.910 | 71400 |
| 36 | 2.490 | 69,2 | 17,2 | 3.900 | 69200 |
| 37 | 2.480 | 67,1 | 17,7 | 3.890 | 67100 |
| 38 | 2.480 | 65,3 | 18,1 | 3.880 | 65300 |
| 39 | 2.480 | 63,5 | 18,6 | 3.880 | 63500 |
| 40 | 2.470 | 61,7 | 19,1 | 3.860 | 61700 |
| 41 | 2.450 | 59,7 | 19,6 | 3.830 | 59700 |
| 42 | 2.420 | 57,6 | 20,1 | 3.790 | 57600 |
| 43 | 2.380 | 55,3 | 20,5 | 3.730 | 55300 |
| 44 | 2.350 | 53,5 | 21 | 3.680 | 53500 |
| 45 | 2.370 | 52,6 | 21,5 | 3.710 | 52600 |
| 46 | 2.380 | 51,7 | 22 | 3.720 | 51700 |
| 47 | 2.370 | 50,5 | 22,4 | 3.720 | 50500 |
| 48 | 2.040 | 42,6 | 22,9 | 3.200 | 42600 |
| 49 | 2.080 | 42,4 | 23,4 | 3.260 | 42400 |
| 50 | 2.110 | 42,2 | 23,9 | 3.300 | 42200 |
| 51 | 2.140 | 41,9 | 24,4 | 3.340 | 41900 |
| 52 | 2.160 | 41,6 | 24,8 | 3.380 | 41600 |
| 53 | 2.190 | 41,3 | 25,3 | 3.420 | 41300 |
| 54 | 2.210 | 40,9 | 25,8 | 3.460 | 40900 |
| 55 | 2.230 | 40,5 | 26,3 | 3.490 | 40500 |
| 56 | 2.240 | 40 | 26,8 | 3.510 | 40000 |
| 57 | 2.250 | 39,5 | 27,2 | 3.520 | 39500 |
| 58 | 2.250 | 38,8 | 27,7 | 3.520 | 38800 |
| 59 | 2.220 | 37,7 | 28,2 | 3.480 | 37700 |
| 60 | 2.180 | 36,3 | 28,7 | 3.410 | 36300 |
| 61 | 2.150 | 35,2 | 29,1 | 3.360 | 35200 |
| 62 | 2.130 | 34,4 | 29,6 | 3.340 | 34400 |
| 63 | 2.130 | 33,9 | 30,1 | 3.340 | 33900 |
| 64 | 2.140 | 33,4 | 30,6 | 3.350 | 33400 |
| 65 | 2.140 | 32,9 | 31 | 3.350 | 32900 |
| 66 | 2.130 | 32,2 | 31,5 | 3.330 | 32200 |
| 67 | 2.110 | 31,4 | 32 | 3.300 | 31400 |
| 68 | 2.080 | 30,7 | 32,5 | 3.260 | 30700 |
| 69 | 2.060 | 29,9 | 33 | 3.230 | 29900 |
| 70 | 2.030 | 29 | 33,4 | 3.180 | 29000 |
| 71 | 2.000 | 28,1 | 33,9 | 3.130 | 28100 |
| 72 | 1.980 | 27,5 | 34,4 | 3.100 | 27500 |
| 73 | 1.960 | 26,9 | 34,9 | 3.070 | 26900 |
| 74 | 1.940 | 26,2 | 35,3 | 3.030 | 26200 |
| 75 | 1.920 | 25,6 | 35,8 | 3.000 | 25600 |
| 76 | 2.200 | 29 | 36,3 | 3.450 | 29000 |
| 77 | 2.180 | 28,4 | 36,8 | 3.420 | 28400 |
| 78 | 2.300 | 29,5 | 37,3 | 3.600 | 29500 |
| 79 | 2.230 | 28,2 | 37,7 | 3.490 | 28200 |
| 80 | 2.340 | 29,3 | 38,2 | 3.660 | 29300 |
| 81 | 2.350 | 29 | 38,7 | 3.680 | 29000 |
| 82 | 2.400 | 29,3 | 39,2 | 3.760 | 29300 |
| 83 | 2.430 | 29,3 | 39,6 | 3.810 | 29300 |
| 84 | 2.480 | 29,5 | 40,1 | 3.880 | 29500 |
| 85 | 2.460 | 29 | 40,6 | 3.850 | 29000 |
| 86 | 2.480 | 28,8 | 41,1 | 3.880 | 28800 |
| 87 | 2.520 | 28,9 | 41,6 | 3.940 | 28900 |
| 88 | 2.500 | 28,4 | 42 | 3.910 | 28400 |
| 89 | 2.530 | 28,4 | 42,5 | 3.950 | 28400 |
| 90 | 2.560 | 28,4 | 43 | 4.000 | 28400 |
| 91 | 2.560 | 28,1 | 43,5 | 4.010 | 28100 |
| 92 | 2.570 | 28 | 43,9 | 4.030 | 28000 |
| 93 | 2.550 | 27,4 | 44,4 | 3.990 | 27400 |
| 94 | 2.570 | 27,4 | 44,9 | 4.030 | 27400 |
| 95 | 2.520 | 26,5 | 45,4 | 3.940 | 26500 |
| 96 | 2.520 | 26,3 | 45,9 | 3.950 | 26300 |
| 97 | 2.540 | 26,2 | 46,3 | 3.970 | 26200 |
| 98 | 2.540 | 25,9 | 46,8 | 3.970 | 25900 |
| 99 | 2.480 | 25,1 | 47,3 | 3.890 | 25100 |
| 100 | 2.490 | 24,9 | 47,8 | 3.890 | 24900 |

**Table 6.** Rheological data of an extra heavy crude oil at 323.15 K (50°C), 1-100 s^-1^, 0.17 T

| Shear Rate | Shear Stress | Viscosity | Speed | Torque | Viscosity |
| --- | --- | --- | --- | --- | --- |
| [1/s] | [Pa] | [Pa·s] | [1/min] | [µNm] | Cp |
| 1 | 55,9 | 55,9 | 0,478 | 87,6 | 55900 |
| 2 | 111 | 55,7 | 0,955 | 174 | 55700 |
| 3 | 167 | 55,5 | 1,43 | 261 | 55500 |
| 4 | 223 | 55,8 | 1,91 | 349 | 55800 |
| 5 | 278 | 55,7 | 2,39 | 436 | 55700 |
| 6 | 334 | 55,6 | 2,87 | 522 | 55600 |
| 7 | 389 | 55,5 | 3,34 | 609 | 55500 |
| 8 | 444 | 55,5 | 3,82 | 695 | 55500 |
| 9 | 499 | 55,4 | 4,3 | 781 | 55400 |
| 10 | 553 | 55,2 | 4,78 | 865 | 55200 |
| 11 | 606 | 55,1 | 5,25 | 949 | 55100 |
| 12 | 660 | 55 | 5,73 | 1.030 | 55000 |
| 13 | 713 | 54,8 | 6,21 | 1.120 | 54800 |
| 14 | 766 | 54,7 | 6,69 | 1.200 | 54700 |
| 15 | 818 | 54,6 | 7,16 | 1.280 | 54600 |
| 16 | 871 | 54,4 | 7,64 | 1.360 | 54400 |
| 17 | 923 | 54,3 | 8,12 | 1.450 | 54300 |
| 18 | 975 | 54,2 | 8,6 | 1.530 | 54200 |
| 19 | 1.030 | 54 | 9,08 | 1.610 | 54000 |
| 20 | 1.080 | 53,8 | 9,55 | 1.690 | 53800 |
| 21 | 1.130 | 53,7 | 10 | 1.760 | 53700 |
| 22 | 1.180 | 53,5 | 10,5 | 1.840 | 53500 |
| 23 | 1.230 | 53,4 | 11 | 1.920 | 53400 |
| 24 | 1.280 | 53,2 | 11,5 | 2.000 | 53200 |
| 25 | 1.330 | 53,1 | 11,9 | 2.080 | 53100 |
| 26 | 1.380 | 52,9 | 12,4 | 2.150 | 52900 |
| 27 | 1.430 | 52,8 | 12,9 | 2.230 | 52800 |
| 28 | 1.470 | 52,7 | 13,4 | 2.310 | 52700 |
| 29 | 1.520 | 52,5 | 13,9 | 2.380 | 52500 |
| 30 | 1.570 | 52,3 | 14,3 | 2.460 | 52300 |
| 31 | 1.620 | 52,2 | 14,8 | 2.530 | 52200 |
| 32 | 1.660 | 52 | 15,3 | 2.610 | 52000 |
| 33 | 1.710 | 51,9 | 15,8 | 2.680 | 51900 |
| 34 | 1.760 | 51,8 | 16,2 | 2.760 | 51800 |
| 35 | 1.810 | 51,6 | 16,7 | 2.830 | 51600 |
| 36 | 1.850 | 51,5 | 17,2 | 2.900 | 51500 |
| 37 | 1.900 | 51,3 | 17,7 | 2.970 | 51300 |
| 38 | 1.940 | 51,2 | 18,2 | 3.040 | 51200 |
| 39 | 1.990 | 51 | 18,6 | 3.110 | 51000 |
| 40 | 2.030 | 50,9 | 19,1 | 3.190 | 50900 |
| 41 | 2.080 | 50,7 | 19,6 | 3.260 | 50700 |
| 42 | 2.120 | 50,6 | 20,1 | 3.330 | 50600 |
| 43 | 2.170 | 50,5 | 20,5 | 3.400 | 50500 |
| 44 | 2.210 | 50,3 | 21 | 3.470 | 50300 |
| 45 | 2.260 | 50,2 | 21,5 | 3.540 | 50200 |
| 46 | 2.300 | 50,1 | 22 | 3.610 | 50100 |
| 47 | 2.350 | 49,9 | 22,4 | 3.670 | 49900 |
| 48 | 2.390 | 49,8 | 22,9 | 3.740 | 49800 |
| 49 | 2.430 | 49,6 | 23,4 | 3.810 | 49600 |
| 50 | 2.470 | 49,4 | 23,9 | 3.870 | 49400 |
| 51 | 2.510 | 49,2 | 24,4 | 3.930 | 49200 |
| 52 | 2.550 | 49 | 24,8 | 3.990 | 49000 |
| 53 | 2.580 | 48,7 | 25,3 | 4.040 | 48700 |
| 54 | 2.620 | 48,4 | 25,8 | 4.100 | 48400 |
| 55 | 2.650 | 48,2 | 26,3 | 4.150 | 48200 |
| 56 | 2.680 | 47,8 | 26,7 | 4.190 | 47800 |
| 57 | 2.700 | 47,4 | 27,2 | 4.230 | 47400 |
| 58 | 2.720 | 46,9 | 27,7 | 4.260 | 46900 |
| 59 | 2.740 | 46,4 | 28,2 | 4.280 | 46400 |
| 60 | 2.740 | 45,7 | 28,7 | 4.300 | 45700 |
| 61 | 2.730 | 44,8 | 29,1 | 4.280 | 44800 |
| 62 | 2.710 | 43,6 | 29,6 | 4.230 | 43600 |
| 63 | 2.650 | 42,1 | 30,1 | 4.150 | 42100 |
| 64 | 2.530 | 39,6 | 30,6 | 3.970 | 39600 |
| 65 | 2.340 | 36 | 31,1 | 3.670 | 36000 |
| 66 | 2.300 | 34,9 | 31,5 | 3.600 | 34900 |
| 67 | 2.270 | 33,9 | 32 | 3.560 | 33900 |
| 68 | 2.240 | 32,9 | 32,5 | 3.500 | 32900 |
| 69 | 2.190 | 31,7 | 33 | 3.420 | 31700 |
| 70 | 2.150 | 30,6 | 33,4 | 3.360 | 30600 |
| 71 | 2.380 | 33,5 | 33,9 | 3.720 | 33500 |
| 72 | 2.330 | 32,3 | 34,4 | 3.640 | 32300 |
| 73 | 2.430 | 33,3 | 34,9 | 3.800 | 33300 |
| 74 | 2.460 | 33,2 | 35,4 | 3.850 | 33200 |
| 75 | 2.500 | 33,4 | 35,8 | 3.920 | 33400 |
| 76 | 2.510 | 33 | 36,3 | 3.920 | 33000 |
| 77 | 2.510 | 32,6 | 36,8 | 3.930 | 32600 |
| 78 | 2.480 | 31,8 | 37,3 | 3.880 | 31800 |
| 79 | 2.500 | 31,6 | 37,7 | 3.910 | 31600 |
| 80 | 2.560 | 32 | 38,2 | 4.000 | 32000 |
| 81 | 2.500 | 30,9 | 38,7 | 3.920 | 30900 |
| 82 | 2.580 | 31,5 | 39,2 | 4.040 | 31500 |
| 83 | 2.570 | 31 | 39,7 | 4.020 | 31000 |
| 84 | 2.510 | 29,9 | 40,1 | 3.930 | 29900 |
| 85 | 2.530 | 29,8 | 40,6 | 3.970 | 29800 |
| 86 | 2.480 | 28,9 | 41,1 | 3.880 | 28900 |
| 87 | 2.550 | 29,3 | 41,6 | 4.000 | 29300 |
| 88 | 2.520 | 28,7 | 42 | 3.950 | 28700 |
| 89 | 2.490 | 28 | 42,5 | 3.900 | 28000 |
| 90 | 2.500 | 27,8 | 43 | 3.920 | 27800 |
| 91 | 2.490 | 27,4 | 43,5 | 3.900 | 27400 |
| 92 | 2.510 | 27,3 | 43,9 | 3.930 | 27300 |
| 93 | 2.440 | 26,2 | 44,4 | 3.820 | 26200 |
| 94 | 2.440 | 26 | 44,9 | 3.820 | 26000 |
| 95 | 2.340 | 24,6 | 45,4 | 3.660 | 24600 |
| 96 | 2.390 | 24,9 | 45,9 | 3.740 | 24900 |
| 97 | 2.390 | 24,7 | 46,3 | 3.750 | 24700 |
| 98 | 2.380 | 24,3 | 46,8 | 3.730 | 24300 |
| 99 | 2.330 | 23,6 | 47,3 | 3.650 | 23600 |
| 100 | 2.300 | 23 | 47,8 | 3.610 | 23000 |

**Table 7.** Rheological data of an extra heavy crude oil at 323.15 K (50°C), 1-100 s^-1^, 0.35 T

| Shear Rate | Shear Stress | Viscosity | Speed | Torque | Viscosity |
| --- | --- | --- | --- | --- | --- |
| [1/s] | [Pa] | [Pa·s] | [1/min] | [µNm] | Cp |
| 1 | 61 | 61 | 0,477 | 95,5 | 61000 |
| 2 | 122 | 60,8 | 0,955 | 191 | 60800 |
| 3 | 182 | 60,6 | 1,43 | 285 | 60600 |
| 4 | 243 | 60,7 | 1,91 | 380 | 60700 |
| 5 | 303 | 60,5 | 2,39 | 474 | 60500 |
| 6 | 362 | 60,4 | 2,87 | 567 | 60400 |
| 7 | 421 | 60,2 | 3,34 | 659 | 60200 |
| 8 | 479 | 59,9 | 3,82 | 750 | 59900 |
| 9 | 537 | 59,7 | 4,3 | 841 | 59700 |
| 10 | 595 | 59,5 | 4,78 | 932 | 59500 |
| 11 | 652 | 59,3 | 5,25 | 1.020 | 59300 |
| 12 | 709 | 59 | 5,73 | 1.110 | 59000 |
| 13 | 764 | 58,8 | 6,21 | 1.200 | 58800 |
| 14 | 820 | 58,6 | 6,69 | 1.280 | 58600 |
| 15 | 875 | 58,4 | 7,16 | 1.370 | 58400 |
| 16 | 929 | 58,1 | 7,64 | 1.460 | 58100 |
| 17 | 983 | 57,8 | 8,12 | 1.540 | 57800 |
| 18 | 1.040 | 57,6 | 8,6 | 1.620 | 57600 |
| 19 | 1.090 | 57,3 | 9,07 | 1.710 | 57300 |
| 20 | 1.140 | 57,1 | 9,55 | 1.790 | 57100 |
| 21 | 1.190 | 56,8 | 10 | 1.870 | 56800 |
| 22 | 1.250 | 56,6 | 10,5 | 1.950 | 56600 |
| 23 | 1.300 | 56,4 | 11 | 2.030 | 56400 |
| 24 | 1.350 | 56,2 | 11,5 | 2.110 | 56200 |
| 25 | 1.400 | 56 | 11,9 | 2.190 | 56000 |
| 26 | 1.450 | 55,8 | 12,4 | 2.270 | 55800 |
| 27 | 1.500 | 55,6 | 12,9 | 2.350 | 55600 |
| 28 | 1.550 | 55,4 | 13,4 | 2.430 | 55400 |
| 29 | 1.600 | 55,1 | 13,8 | 2.500 | 55100 |
| 30 | 1.650 | 54,9 | 14,3 | 2.580 | 54900 |
| 31 | 1.700 | 54,7 | 14,8 | 2.650 | 54700 |
| 32 | 1.740 | 54,5 | 15,3 | 2.730 | 54500 |
| 33 | 1.790 | 54,3 | 15,8 | 2.810 | 54300 |
| 34 | 1.840 | 54,1 | 16,2 | 2.880 | 54100 |
| 35 | 1.890 | 53,9 | 16,7 | 2.950 | 53900 |
| 36 | 1.930 | 53,7 | 17,2 | 3.020 | 53700 |
| 37 | 1.980 | 53,5 | 17,7 | 3.100 | 53500 |
| 38 | 2.020 | 53,2 | 18,2 | 3.170 | 53200 |
| 39 | 2.070 | 53,1 | 18,6 | 3.240 | 53100 |
| 40 | 2.110 | 52,8 | 19,1 | 3.310 | 52800 |
| 41 | 2.160 | 52,6 | 19,6 | 3.380 | 52600 |
| 42 | 2.200 | 52,4 | 20,1 | 3.450 | 52400 |
| 43 | 2.250 | 52,3 | 20,5 | 3.520 | 52300 |
| 44 | 2.290 | 52 | 21 | 3.590 | 52000 |
| 45 | 2.330 | 51,8 | 21,5 | 3.650 | 51800 |
| 46 | 2.370 | 51,6 | 22 | 3.720 | 51600 |
| 47 | 2.420 | 51,4 | 22,4 | 3.780 | 51400 |
| 48 | 2.460 | 51,2 | 22,9 | 3.850 | 51200 |
| 49 | 2.500 | 51 | 23,4 | 3.910 | 51000 |
| 50 | 2.540 | 50,7 | 23,9 | 3.970 | 50700 |
| 51 | 2.570 | 50,5 | 24,4 | 4.030 | 50500 |
| 52 | 2.610 | 50,3 | 24,8 | 4.090 | 50300 |
| 53 | 2.650 | 50 | 25,3 | 4.150 | 50000 |
| 54 | 2.690 | 49,8 | 25,8 | 4.210 | 49800 |
| 55 | 2.730 | 49,6 | 26,3 | 4.270 | 49600 |
| 56 | 2.760 | 49,3 | 26,7 | 4.320 | 49300 |
| 57 | 2.800 | 49,1 | 27,2 | 4.380 | 49100 |
| 58 | 2.830 | 48,8 | 27,7 | 4.430 | 48800 |
| 59 | 2.860 | 48,5 | 28,2 | 4.480 | 48500 |
| 60 | 2.900 | 48,3 | 28,6 | 4.530 | 48300 |
| 61 | 2.930 | 48 | 29,1 | 4.580 | 48000 |
| 62 | 2.960 | 47,7 | 29,6 | 4.630 | 47700 |
| 63 | 2.990 | 47,4 | 30,1 | 4.670 | 47400 |
| 64 | 3.010 | 47 | 30,6 | 4.710 | 47000 |
| 65 | 3.020 | 46,5 | 31 | 4.730 | 46500 |
| 66 | 3.030 | 46 | 31,5 | 4.750 | 46000 |
| 67 | 3.030 | 45,2 | 32 | 4.740 | 45200 |
| 68 | 2.990 | 44 | 32,5 | 4.690 | 44000 |
| 69 | 2.920 | 42,4 | 32,9 | 4.580 | 42400 |
| 70 | 2.800 | 39,9 | 33,4 | 4.380 | 39900 |
| 71 | 2.570 | 36,2 | 33,9 | 4.020 | 36200 |
| 72 | 2.410 | 33,4 | 34,4 | 3.770 | 33400 |
| 73 | 2.550 | 35 | 34,9 | 3.990 | 35000 |
| 74 | 2.410 | 32,5 | 35,3 | 3.770 | 32500 |
| 75 | 2.460 | 32,7 | 35,8 | 3.840 | 32700 |
| 76 | 2.460 | 32,3 | 36,3 | 3.850 | 32300 |
| 77 | 2.500 | 32,5 | 36,8 | 3.910 | 32500 |
| 78 | 2.560 | 32,8 | 37,3 | 4.000 | 32800 |
| 79 | 2.520 | 32 | 37,7 | 3.950 | 32000 |
| 80 | 2.500 | 31,2 | 38,2 | 3.910 | 31200 |
| 81 | 2.570 | 31,7 | 38,7 | 4.020 | 31700 |
| 82 | 2.560 | 31,2 | 39,2 | 4.010 | 31200 |
| 83 | 2.590 | 31,2 | 39,6 | 4.050 | 31200 |
| 84 | 2.510 | 29,9 | 40,1 | 3.930 | 29900 |
| 85 | 2.540 | 29,9 | 40,6 | 3.980 | 29900 |
| 86 | 2.550 | 29,6 | 41,1 | 3.990 | 29600 |
| 87 | 2.560 | 29,4 | 41,5 | 4.000 | 29400 |
| 88 | 2.560 | 29,1 | 42 | 4.010 | 29100 |
| 89 | 2.540 | 28,5 | 42,5 | 3.980 | 28500 |
| 90 | 2.520 | 28 | 43 | 3.940 | 28000 |
| 91 | 2.480 | 27,3 | 43,5 | 3.880 | 27300 |
| 92 | 2.500 | 27,2 | 43,9 | 3.920 | 27200 |
| 93 | 2.460 | 26,4 | 44,4 | 3.850 | 26400 |
| 94 | 2.500 | 26,6 | 44,9 | 3.910 | 26600 |
| 95 | 2.490 | 26,2 | 45,4 | 3.900 | 26200 |
| 96 | 2.490 | 25,9 | 45,9 | 3.900 | 25900 |
| 97 | 2.400 | 24,8 | 46,3 | 3.760 | 24800 |
| 98 | 2.400 | 24,5 | 46,8 | 3.760 | 24500 |
| 99 | 2.430 | 24,6 | 47,3 | 3.810 | 24600 |
| 100 | 2.480 | 24,8 | 47,8 | 3.880 | 24800 |

**Table 8.** Rheological data of an extra heavy crude oil at 323.15 K (50°C), 1-100 s^-1^, 0.65 T

| Shear Rate | Shear Stress | Viscosity | Speed | Torque | Viscosity |
| --- | --- | --- | --- | --- | --- |
| [1/s] | [Pa] | [Pa·s] | [1/min] | [µNm] | Cp |
| 1 | 56,6 | 56,6 | 0,477 | 88,6 | 56600 |
| 2 | 114 | 56,8 | 0,955 | 178 | 56800 |
| 3 | 170 | 56,7 | 1,43 | 266 | 56700 |
| 4 | 227 | 56,7 | 1,91 | 355 | 56700 |
| 5 | 282 | 56,4 | 2,39 | 442 | 56400 |
| 6 | 337 | 56,2 | 2,87 | 528 | 56200 |
| 7 | 391 | 55,8 | 3,34 | 611 | 55800 |
| 8 | 443 | 55,4 | 3,82 | 693 | 55400 |
| 9 | 494 | 54,9 | 4,3 | 773 | 54900 |
| 10 | 544 | 54,4 | 4,78 | 851 | 54400 |
| 11 | 592 | 53,8 | 5,25 | 927 | 53800 |
| 12 | 640 | 53,3 | 5,73 | 1.000 | 53300 |
| 13 | 687 | 52,8 | 6,21 | 1.070 | 52800 |
| 14 | 732 | 52,3 | 6,68 | 1.150 | 52300 |
| 15 | 775 | 51,7 | 7,16 | 1.210 | 51700 |
| 16 | 818 | 51,1 | 7,64 | 1.280 | 51100 |
| 17 | 861 | 50,6 | 8,12 | 1.350 | 50600 |
| 18 | 902 | 50,1 | 8,6 | 1.410 | 50100 |
| 19 | 942 | 49,6 | 9,07 | 1.470 | 49600 |
| 20 | 981 | 49,1 | 9,55 | 1.540 | 49100 |
| 21 | 1.020 | 48,6 | 10 | 1.600 | 48600 |
| 22 | 1.060 | 48,1 | 10,5 | 1.660 | 48100 |
| 23 | 1.100 | 47,6 | 11 | 1.720 | 47600 |
| 24 | 1.130 | 47,2 | 11,5 | 1.770 | 47200 |
| 25 | 1.170 | 46,8 | 11,9 | 1.830 | 46800 |
| 26 | 1.210 | 46,4 | 12,4 | 1.890 | 46400 |
| 27 | 1.240 | 45,9 | 12,9 | 1.940 | 45900 |
| 28 | 1.270 | 45,5 | 13,4 | 2.000 | 45500 |
| 29 | 1.310 | 45,1 | 13,8 | 2.050 | 45100 |
| 30 | 1.340 | 44,7 | 14,3 | 2.100 | 44700 |
| 31 | 1.380 | 44,4 | 14,8 | 2.150 | 44400 |
| 32 | 1.410 | 44 | 15,3 | 2.200 | 44000 |
| 33 | 1.440 | 43,6 | 15,8 | 2.260 | 43600 |
| 34 | 1.470 | 43,3 | 16,2 | 2.300 | 43300 |
| 35 | 1.500 | 42,9 | 16,7 | 2.350 | 42900 |
| 36 | 1.530 | 42,6 | 17,2 | 2.400 | 42600 |
| 37 | 1.560 | 42,3 | 17,7 | 2.450 | 42300 |
| 38 | 1.600 | 42 | 18,1 | 2.500 | 42000 |
| 39 | 1.630 | 41,7 | 18,6 | 2.540 | 41700 |
| 40 | 1.650 | 41,4 | 19,1 | 2.590 | 41400 |
| 41 | 1.690 | 41,1 | 19,6 | 2.640 | 41100 |
| 42 | 1.710 | 40,8 | 20,1 | 2.680 | 40800 |
| 43 | 1.740 | 40,6 | 20,5 | 2.730 | 40600 |
| 44 | 1.770 | 40,3 | 21 | 2.780 | 40300 |
| 45 | 1.800 | 40 | 21,5 | 2.820 | 40000 |
| 46 | 1.830 | 39,8 | 22 | 2.860 | 39800 |
| 47 | 1.860 | 39,5 | 22,4 | 2.910 | 39500 |
| 48 | 1.890 | 39,3 | 22,9 | 2.950 | 39300 |
| 49 | 1.910 | 39 | 23,4 | 2.990 | 39000 |
| 50 | 1.940 | 38,8 | 23,9 | 3.040 | 38800 |
| 51 | 1.970 | 38,6 | 24,4 | 3.080 | 38600 |
| 52 | 2.000 | 38,4 | 24,8 | 3.120 | 38400 |
| 53 | 2.020 | 38,2 | 25,3 | 3.160 | 38200 |
| 54 | 2.050 | 37,9 | 25,8 | 3.210 | 37900 |
| 55 | 2.070 | 37,7 | 26,3 | 3.250 | 37700 |
| 56 | 2.100 | 37,5 | 26,7 | 3.290 | 37500 |
| 57 | 2.130 | 37,3 | 27,2 | 3.330 | 37300 |
| 58 | 2.150 | 37,1 | 27,7 | 3.370 | 37100 |
| 59 | 2.180 | 36,9 | 28,2 | 3.410 | 36900 |
| 60 | 2.210 | 36,8 | 28,6 | 3.450 | 36800 |
| 61 | 2.230 | 36,6 | 29,1 | 3.490 | 36600 |
| 62 | 2.250 | 36,4 | 29,6 | 3.530 | 36400 |
| 63 | 2.280 | 36,2 | 30,1 | 3.570 | 36200 |
| 64 | 2.300 | 36 | 30,6 | 3.610 | 36000 |
| 65 | 2.330 | 35,8 | 31 | 3.650 | 35800 |
| 66 | 2.350 | 35,7 | 31,5 | 3.690 | 35700 |
| 67 | 2.380 | 35,5 | 32 | 3.720 | 35500 |
| 68 | 2.400 | 35,3 | 32,5 | 3.760 | 35300 |
| 69 | 2.430 | 35,2 | 33 | 3.800 | 35200 |
| 70 | 2.450 | 35 | 33,4 | 3.840 | 35000 |
| 71 | 2.480 | 34,9 | 33,9 | 3.880 | 34900 |
| 72 | 2.500 | 34,7 | 34,4 | 3.910 | 34700 |
| 73 | 2.520 | 34,6 | 34,9 | 3.950 | 34600 |
| 74 | 2.540 | 34,4 | 35,3 | 3.980 | 34400 |
| 75 | 2.570 | 34,3 | 35,8 | 4.020 | 34300 |
| 76 | 2.590 | 34,1 | 36,3 | 4.060 | 34100 |
| 77 | 2.610 | 33,9 | 36,8 | 4.090 | 33900 |
| 78 | 2.640 | 33,8 | 37,2 | 4.130 | 33800 |
| 79 | 2.660 | 33,7 | 37,7 | 4.160 | 33700 |
| 80 | 2.680 | 33,5 | 38,2 | 4.200 | 33500 |
| 81 | 2.700 | 33,4 | 38,7 | 4.230 | 33400 |
| 82 | 2.730 | 33,3 | 39,1 | 4.270 | 33300 |
| 83 | 2.750 | 33,1 | 39,6 | 4.310 | 33100 |
| 84 | 2.770 | 33 | 40,1 | 4.340 | 33000 |
| 85 | 2.800 | 32,9 | 40,6 | 4.380 | 32900 |
| 86 | 2.820 | 32,8 | 41,1 | 4.410 | 32800 |
| 87 | 2.840 | 32,7 | 41,5 | 4.450 | 32700 |
| 88 | 2.870 | 32,6 | 42 | 4.490 | 32600 |
| 89 | 2.890 | 32,5 | 42,5 | 4.520 | 32500 |
| 90 | 2.910 | 32,4 | 43 | 4.560 | 32400 |
| 91 | 2.940 | 32,3 | 43,5 | 4.600 | 32300 |
| 92 | 2.960 | 32,2 | 43,9 | 4.640 | 32200 |
| 93 | 2.990 | 32,1 | 44,4 | 4.670 | 32100 |
| 94 | 3.010 | 32 | 44,9 | 4.710 | 32000 |
| 95 | 3.030 | 31,9 | 45,4 | 4.740 | 31900 |
| 96 | 3.050 | 31,8 | 45,8 | 4.780 | 31800 |
| 97 | 3.070 | 31,7 | 46,3 | 4.810 | 31700 |
| 98 | 3.090 | 31,5 | 46,8 | 4.840 | 31500 |
| 99 | 3.110 | 31,4 | 47,3 | 4.870 | 31400 |
| 100 | 3.120 | 31,3 | 47,7 | 4.890 | 31300 |

**Table 9.** Rheological data of an extra heavy crude oil at 343.15 K (70°C), 1-100 s^-1^

| Shear Rate | Shear Stress | Viscosity | Speed | Torque | Viscosity |
| --- | --- | --- | --- | --- | --- |
| [1/s] | [Pa] | [Pa·s] | [1/min] | [µNm] | Cp |
| 1 | 22,9 | 22,9 | 0,478 | 35,8 | 22900 |
| 2 | 44,9 | 22,5 | 0,956 | 70,4 | 22500 |
| 3 | 65,7 | 21,9 | 1,43 | 103 | 21900 |
| 4 | 87,3 | 21,8 | 1,91 | 137 | 21800 |
| 5 | 109 | 21,8 | 2,39 | 170 | 21800 |
| 6 | 129 | 21,6 | 2,87 | 202 | 21600 |
| 7 | 149 | 21,3 | 3,35 | 234 | 21300 |
| 8 | 170 | 21,2 | 3,82 | 266 | 21200 |
| 9 | 191 | 21,2 | 4,3 | 299 | 21200 |
| 10 | 211 | 21,1 | 4,78 | 330 | 21100 |
| 11 | 230 | 20,9 | 5,26 | 360 | 20900 |
| 12 | 248 | 20,7 | 5,74 | 388 | 20700 |
| 13 | 268 | 20,6 | 6,21 | 420 | 20600 |
| 14 | 285 | 20,4 | 6,69 | 446 | 20400 |
| 15 | 302 | 20,1 | 7,17 | 472 | 20100 |
| 16 | 315 | 19,7 | 7,65 | 494 | 19700 |
| 17 | 331 | 19,4 | 8,13 | 517 | 19400 |
| 18 | 346 | 19,2 | 8,6 | 542 | 19200 |
| 19 | 363 | 19,1 | 9,08 | 568 | 19100 |
| 20 | 380 | 19 | 9,56 | 595 | 19000 |
| 21 | 396 | 18,8 | 10 | 620 | 18800 |
| 22 | 414 | 18,8 | 10,5 | 649 | 18800 |
| 23 | 429 | 18,7 | 11 | 672 | 18700 |
| 24 | 445 | 18,6 | 11,5 | 697 | 18600 |
| 25 | 465 | 18,6 | 11,9 | 727 | 18600 |
| 26 | 484 | 18,6 | 12,4 | 757 | 18600 |
| 27 | 500 | 18,5 | 12,9 | 783 | 18500 |
| 28 | 519 | 18,5 | 13,4 | 812 | 18500 |
| 29 | 536 | 18,5 | 13,9 | 839 | 18500 |
| 30 | 552 | 18,4 | 14,3 | 865 | 18400 |
| 31 | 575 | 18,6 | 14,8 | 900 | 18600 |
| 32 | 595 | 18,6 | 15,3 | 932 | 18600 |
| 33 | 619 | 18,7 | 15,8 | 968 | 18700 |
| 34 | 640 | 18,8 | 16,3 | 1.000 | 18800 |
| 35 | 654 | 18,7 | 16,7 | 1.020 | 18700 |
| 36 | 670 | 18,6 | 17,2 | 1.050 | 18600 |
| 37 | 688 | 18,6 | 17,7 | 1.080 | 18600 |
| 38 | 710 | 18,7 | 18,2 | 1.110 | 18700 |
| 39 | 731 | 18,7 | 18,6 | 1.140 | 18700 |
| 40 | 749 | 18,7 | 19,1 | 1.170 | 18700 |
| 41 | 766 | 18,7 | 19,6 | 1.200 | 18700 |
| 42 | 783 | 18,6 | 20,1 | 1.230 | 18600 |
| 43 | 803 | 18,7 | 20,6 | 1.260 | 18700 |
| 44 | 827 | 18,8 | 21 | 1.290 | 18800 |
| 45 | 850 | 18,9 | 21,5 | 1.330 | 18900 |
| 46 | 865 | 18,8 | 22 | 1.350 | 18800 |
| 47 | 885 | 18,8 | 22,5 | 1.390 | 18800 |
| 48 | 905 | 18,9 | 22,9 | 1.420 | 18900 |
| 49 | 917 | 18,7 | 23,4 | 1.440 | 18700 |
| 50 | 934 | 18,7 | 23,9 | 1.460 | 18700 |
| 51 | 957 | 18,8 | 24,4 | 1.500 | 18800 |
| 52 | 975 | 18,7 | 24,9 | 1.530 | 18700 |
| 53 | 992 | 18,7 | 25,3 | 1.550 | 18700 |
| 54 | 1.010 | 18,7 | 25,8 | 1.580 | 18700 |
| 55 | 1.030 | 18,7 | 26,3 | 1.610 | 18700 |
| 56 | 1.050 | 18,7 | 26,8 | 1.640 | 18700 |
| 57 | 1.060 | 18,6 | 27,2 | 1.660 | 18600 |
| 58 | 1.080 | 18,6 | 27,7 | 1.690 | 18600 |
| 59 | 1.100 | 18,7 | 28,2 | 1.730 | 18700 |
| 60 | 1.140 | 19 | 28,7 | 1.790 | 19000 |
| 61 | 1.160 | 19 | 29,2 | 1.820 | 19000 |
| 62 | 1.180 | 19 | 29,6 | 1.850 | 19000 |
| 63 | 1.200 | 19 | 30,1 | 1.880 | 19000 |
| 64 | 1.220 | 19,1 | 30,6 | 1.920 | 19100 |
| 65 | 1.250 | 19,2 | 31,1 | 1.950 | 19200 |
| 66 | 1.260 | 19,1 | 31,5 | 1.970 | 19100 |
| 67 | 1.280 | 19 | 32 | 2.000 | 19000 |
| 68 | 1.300 | 19,1 | 32,5 | 2.030 | 19100 |
| 69 | 1.320 | 19,1 | 33 | 2.070 | 19100 |
| 70 | 1.350 | 19,2 | 33,5 | 2.110 | 19200 |
| 71 | 1.370 | 19,2 | 33,9 | 2.140 | 19200 |
| 72 | 1.380 | 19,2 | 34,4 | 2.170 | 19200 |
| 73 | 1.400 | 19,2 | 34,9 | 2.200 | 19200 |
| 74 | 1.420 | 19,2 | 35,4 | 2.220 | 19200 |
| 75 | 1.440 | 19,2 | 35,8 | 2.250 | 19200 |
| 76 | 1.460 | 19,2 | 36,3 | 2.290 | 19200 |
| 77 | 1.470 | 19,1 | 36,8 | 2.300 | 19100 |
| 78 | 1.490 | 19,1 | 37,3 | 2.330 | 19100 |
| 79 | 1.500 | 19,1 | 37,8 | 2.360 | 19100 |
| 80 | 1.520 | 19 | 38,2 | 2.380 | 19000 |
| 81 | 1.540 | 19 | 38,7 | 2.410 | 19000 |
| 82 | 1.560 | 19 | 39,2 | 2.440 | 19000 |
| 83 | 1.590 | 19,1 | 39,7 | 2.490 | 19100 |
| 84 | 1.620 | 19,3 | 40,1 | 2.540 | 19300 |
| 85 | 1.640 | 19,3 | 40,6 | 2.560 | 19300 |
| 86 | 1.650 | 19,2 | 41,1 | 2.580 | 19200 |
| 87 | 1.670 | 19,2 | 41,6 | 2.620 | 19200 |
| 88 | 1.700 | 19,3 | 42,1 | 2.660 | 19300 |
| 89 | 1.710 | 19,2 | 42,5 | 2.680 | 19200 |
| 90 | 1.730 | 19,2 | 43 | 2.700 | 19200 |
| 91 | 1.750 | 19,2 | 43,5 | 2.730 | 19200 |
| 92 | 1.760 | 19,2 | 44 | 2.760 | 19200 |
| 93 | 1.780 | 19,2 | 44,5 | 2.790 | 19200 |
| 94 | 1.790 | 19,1 | 44,9 | 2.810 | 19100 |
| 95 | 1.820 | 19,1 | 45,4 | 2.850 | 19100 |
| 96 | 1.840 | 19,2 | 45,9 | 2.890 | 19200 |
| 97 | 1.860 | 19,2 | 46,4 | 2.910 | 19200 |
| 98 | 1.880 | 19,2 | 46,8 | 2.950 | 19200 |
| 99 | 1.900 | 19,2 | 47,3 | 2.980 | 19200 |
| 100 | 1.920 | 19,2 | 47,8 | 3.000 | 19200 |

**Table 10** Rheological data of an extra heavy crude oil at 343.15 K (70°C), 1-100 s^-1^, 0.17 T

| Shear Rate | Shear Stress | Viscosity | Speed | Torque | Viscosity |
| --- | --- | --- | --- | --- | --- |
| [1/s] | [Pa] | [Pa·s] | [1/min] | [µNm] | Cp |
| 1 | 26,5 | 26,5 | 0,478 | 41,5 | 26500 |
| 2 | 52,9 | 26,5 | 0,956 | 82,9 | 26500 |
| 3 | 79 | 26,3 | 1,43 | 124 | 26300 |
| 4 | 104 | 26,1 | 1,91 | 163 | 26100 |
| 5 | 129 | 25,8 | 2,39 | 202 | 25800 |
| 6 | 153 | 25,5 | 2,87 | 239 | 25500 |
| 7 | 176 | 25,1 | 3,35 | 275 | 25100 |
| 8 | 198 | 24,8 | 3,82 | 310 | 24800 |
| 9 | 219 | 24,4 | 4,3 | 344 | 24400 |
| 10 | 240 | 24 | 4,78 | 376 | 24000 |
| 11 | 260 | 23,7 | 5,26 | 407 | 23700 |
| 12 | 280 | 23,3 | 5,74 | 438 | 23300 |
| 13 | 298 | 23 | 6,21 | 467 | 23000 |
| 14 | 317 | 22,6 | 6,69 | 496 | 22600 |
| 15 | 334 | 22,3 | 7,17 | 523 | 22300 |
| 16 | 352 | 22 | 7,65 | 550 | 22000 |
| 17 | 368 | 21,7 | 8,13 | 577 | 21700 |
| 18 | 385 | 21,4 | 8,6 | 603 | 21400 |
| 19 | 401 | 21,1 | 9,08 | 628 | 21100 |
| 20 | 417 | 20,8 | 9,56 | 653 | 20800 |
| 21 | 433 | 20,6 | 10 | 677 | 20600 |
| 22 | 448 | 20,3 | 10,5 | 701 | 20300 |
| 23 | 463 | 20,1 | 11 | 724 | 20100 |
| 24 | 477 | 19,9 | 11,5 | 747 | 19900 |
| 25 | 492 | 19,7 | 11,9 | 769 | 19700 |
| 26 | 506 | 19,5 | 12,4 | 792 | 19500 |
| 27 | 520 | 19,3 | 12,9 | 814 | 19300 |
| 28 | 534 | 19,1 | 13,4 | 835 | 19100 |
| 29 | 547 | 18,9 | 13,9 | 857 | 18900 |
| 30 | 561 | 18,7 | 14,3 | 878 | 18700 |
| 31 | 574 | 18,5 | 14,8 | 898 | 18500 |
| 32 | 587 | 18,3 | 15,3 | 919 | 18300 |
| 33 | 600 | 18,2 | 15,8 | 939 | 18200 |
| 34 | 613 | 18 | 16,3 | 960 | 18000 |
| 35 | 627 | 17,9 | 16,7 | 981 | 17900 |
| 36 | 640 | 17,8 | 17,2 | 1.000 | 17800 |
| 37 | 653 | 17,6 | 17,7 | 1.020 | 17600 |
| 38 | 666 | 17,5 | 18,2 | 1.040 | 17500 |
| 39 | 679 | 17,4 | 18,6 | 1.060 | 17400 |
| 40 | 691 | 17,3 | 19,1 | 1.080 | 17300 |
| 41 | 704 | 17,2 | 19,6 | 1.100 | 17200 |
| 42 | 717 | 17,1 | 20,1 | 1.120 | 17100 |
| 43 | 730 | 17 | 20,6 | 1.140 | 17000 |
| 44 | 743 | 16,9 | 21 | 1.160 | 16900 |
| 45 | 756 | 16,8 | 21,5 | 1.180 | 16800 |
| 46 | 769 | 16,7 | 22 | 1.200 | 16700 |
| 47 | 781 | 16,6 | 22,5 | 1.220 | 16600 |
| 48 | 794 | 16,5 | 22,9 | 1.240 | 16500 |
| 49 | 806 | 16,5 | 23,4 | 1.260 | 16500 |
| 50 | 820 | 16,4 | 23,9 | 1.280 | 16400 |
| 51 | 832 | 16,3 | 24,4 | 1.300 | 16300 |
| 52 | 846 | 16,3 | 24,9 | 1.320 | 16300 |
| 53 | 859 | 16,2 | 25,3 | 1.340 | 16200 |
| 54 | 873 | 16,2 | 25,8 | 1.370 | 16200 |
| 55 | 885 | 16,1 | 26,3 | 1.390 | 16100 |
| 56 | 907 | 16,2 | 26,8 | 1.420 | 16200 |
| 57 | 921 | 16,2 | 27,2 | 1.440 | 16200 |
| 58 | 934 | 16,1 | 27,7 | 1.460 | 16100 |
| 59 | 948 | 16,1 | 28,2 | 1.480 | 16100 |
| 60 | 963 | 16,1 | 28,7 | 1.510 | 16100 |
| 61 | 977 | 16 | 29,2 | 1.530 | 16000 |
| 62 | 990 | 16 | 29,6 | 1.550 | 16000 |
| 63 | 1.000 | 15,9 | 30,1 | 1.570 | 15900 |
| 64 | 1.020 | 15,9 | 30,6 | 1.590 | 15900 |
| 65 | 1.030 | 15,9 | 31,1 | 1.610 | 15900 |
| 66 | 1.050 | 15,8 | 31,5 | 1.640 | 15800 |
| 67 | 1.060 | 15,8 | 32 | 1.660 | 15800 |
| 68 | 1.070 | 15,8 | 32,5 | 1.680 | 15800 |
| 69 | 1.090 | 15,7 | 33 | 1.700 | 15700 |
| 70 | 1.100 | 15,7 | 33,5 | 1.720 | 15700 |
| 71 | 1.110 | 15,7 | 33,9 | 1.740 | 15700 |
| 72 | 1.130 | 15,7 | 34,4 | 1.770 | 15700 |
| 73 | 1.140 | 15,6 | 34,9 | 1.790 | 15600 |
| 74 | 1.160 | 15,6 | 35,4 | 1.810 | 15600 |
| 75 | 1.170 | 15,6 | 35,8 | 1.830 | 15600 |
| 76 | 1.180 | 15,6 | 36,3 | 1.850 | 15600 |
| 77 | 1.200 | 15,5 | 36,8 | 1.870 | 15500 |
| 78 | 1.210 | 15,5 | 37,3 | 1.900 | 15500 |
| 79 | 1.220 | 15,5 | 37,8 | 1.920 | 15500 |
| 80 | 1.240 | 15,5 | 38,2 | 1.940 | 15500 |
| 81 | 1.250 | 15,5 | 38,7 | 1.960 | 15500 |
| 82 | 1.270 | 15,5 | 39,2 | 1.990 | 15500 |
| 83 | 1.280 | 15,4 | 39,7 | 2.010 | 15400 |
| 84 | 1.300 | 15,4 | 40,1 | 2.030 | 15400 |
| 85 | 1.310 | 15,4 | 40,6 | 2.050 | 15400 |
| 86 | 1.320 | 15,4 | 41,1 | 2.070 | 15400 |
| 87 | 1.340 | 15,4 | 41,6 | 2.100 | 15400 |
| 88 | 1.350 | 15,4 | 42,1 | 2.120 | 15400 |
| 89 | 1.370 | 15,4 | 42,5 | 2.140 | 15400 |
| 90 | 1.380 | 15,4 | 43 | 2.160 | 15400 |
| 91 | 1.400 | 15,4 | 43,5 | 2.190 | 15400 |
| 92 | 1.410 | 15,3 | 44 | 2.210 | 15300 |
| 93 | 1.420 | 15,3 | 44,4 | 2.230 | 15300 |
| 94 | 1.440 | 15,3 | 44,9 | 2.260 | 15300 |
| 95 | 1.460 | 15,3 | 45,4 | 2.280 | 15300 |
| 96 | 1.470 | 15,3 | 45,9 | 2.300 | 15300 |
| 97 | 1.480 | 15,3 | 46,4 | 2.320 | 15300 |
| 98 | 1.500 | 15,3 | 46,8 | 2.350 | 15300 |
| 99 | 1.510 | 15,3 | 47,3 | 2.370 | 15300 |
| 100 | 1.530 | 15,3 | 47,8 | 2.390 | 15300 |

**Table 11** Rheological data of an extra heavy crude oil at 343.15 K (70°C), 1-100 s^-1^, 0.35 T

| Shear Rate | Shear Stress | Viscosity | Speed | Torque | Viscosity |
| --- | --- | --- | --- | --- | --- |
| [1/s] | [Pa] | [Pa·s] | [1/min] | [µNm] | Cp |
| 1 | 15,9 | 15,9 | 0,478 | 24,9 | 15900 |
| 2 | 31,8 | 15,9 | 0,956 | 49,9 | 15900 |
| 3 | 47,7 | 15,9 | 1,43 | 74,6 | 15900 |
| 4 | 63,6 | 15,9 | 1,91 | 99,6 | 15900 |
| 5 | 79,4 | 15,9 | 2,39 | 124 | 15900 |
| 6 | 95,2 | 15,9 | 2,87 | 149 | 15900 |
| 7 | 111 | 15,8 | 3,35 | 174 | 15800 |
| 8 | 127 | 15,8 | 3,82 | 198 | 15800 |
| 9 | 142 | 15,8 | 4,3 | 223 | 15800 |
| 10 | 158 | 15,8 | 4,78 | 247 | 15800 |
| 11 | 173 | 15,7 | 5,26 | 271 | 15700 |
| 12 | 188 | 15,7 | 5,74 | 295 | 15700 |
| 13 | 204 | 15,7 | 6,21 | 319 | 15700 |
| 14 | 219 | 15,6 | 6,69 | 342 | 15600 |
| 15 | 234 | 15,6 | 7,17 | 366 | 15600 |
| 16 | 249 | 15,5 | 7,65 | 389 | 15500 |
| 17 | 263 | 15,5 | 8,12 | 412 | 15500 |
| 18 | 278 | 15,5 | 8,6 | 436 | 15500 |
| 19 | 293 | 15,4 | 9,08 | 458 | 15400 |
| 20 | 307 | 15,4 | 9,56 | 481 | 15400 |
| 21 | 322 | 15,3 | 10 | 504 | 15300 |
| 22 | 336 | 15,3 | 10,5 | 526 | 15300 |
| 23 | 350 | 15,2 | 11 | 548 | 15200 |
| 24 | 364 | 15,2 | 11,5 | 570 | 15200 |
| 25 | 378 | 15,1 | 11,9 | 592 | 15100 |
| 26 | 392 | 15,1 | 12,4 | 614 | 15100 |
| 27 | 406 | 15 | 12,9 | 636 | 15000 |
| 28 | 420 | 15 | 13,4 | 658 | 15000 |
| 29 | 434 | 15 | 13,9 | 679 | 15000 |
| 30 | 448 | 14,9 | 14,3 | 701 | 14900 |
| 31 | 462 | 14,9 | 14,8 | 723 | 14900 |
| 32 | 476 | 14,9 | 15,3 | 745 | 14900 |
| 33 | 489 | 14,8 | 15,8 | 766 | 14800 |
| 34 | 503 | 14,8 | 16,2 | 787 | 14800 |
| 35 | 517 | 14,8 | 16,7 | 809 | 14800 |
| 36 | 530 | 14,7 | 17,2 | 830 | 14700 |
| 37 | 544 | 14,7 | 17,7 | 851 | 14700 |
| 38 | 557 | 14,7 | 18,2 | 872 | 14700 |
| 39 | 571 | 14,6 | 18,6 | 894 | 14600 |
| 40 | 584 | 14,6 | 19,1 | 915 | 14600 |
| 41 | 598 | 14,6 | 19,6 | 936 | 14600 |
| 42 | 611 | 14,6 | 20,1 | 957 | 14600 |
| 43 | 626 | 14,6 | 20,5 | 980 | 14600 |
| 44 | 639 | 14,5 | 21 | 1.000 | 14500 |
| 45 | 654 | 14,5 | 21,5 | 1.020 | 14500 |
| 46 | 667 | 14,5 | 22 | 1.040 | 14500 |
| 47 | 681 | 14,5 | 22,5 | 1.070 | 14500 |
| 48 | 694 | 14,5 | 22,9 | 1.090 | 14500 |
| 49 | 707 | 14,4 | 23,4 | 1.110 | 14400 |
| 50 | 720 | 14,4 | 23,9 | 1.130 | 14400 |
| 51 | 734 | 14,4 | 24,4 | 1.150 | 14400 |
| 52 | 747 | 14,4 | 24,9 | 1.170 | 14400 |
| 53 | 761 | 14,4 | 25,3 | 1.190 | 14400 |
| 54 | 774 | 14,3 | 25,8 | 1.210 | 14300 |
| 55 | 787 | 14,3 | 26,3 | 1.230 | 14300 |
| 56 | 809 | 14,4 | 26,8 | 1.270 | 14400 |
| 57 | 824 | 14,5 | 27,2 | 1.290 | 14500 |
| 58 | 838 | 14,4 | 27,7 | 1.310 | 14400 |
| 59 | 852 | 14,4 | 28,2 | 1.330 | 14400 |
| 60 | 866 | 14,4 | 28,7 | 1.360 | 14400 |
| 61 | 880 | 14,4 | 29,2 | 1.380 | 14400 |
| 62 | 894 | 14,4 | 29,6 | 1.400 | 14400 |
| 63 | 908 | 14,4 | 30,1 | 1.420 | 14400 |
| 64 | 921 | 14,4 | 30,6 | 1.440 | 14400 |
| 65 | 935 | 14,4 | 31,1 | 1.460 | 14400 |
| 66 | 949 | 14,4 | 31,5 | 1.490 | 14400 |
| 67 | 964 | 14,4 | 32 | 1.510 | 14400 |
| 68 | 977 | 14,4 | 32,5 | 1.530 | 14400 |
| 69 | 990 | 14,4 | 33 | 1.550 | 14400 |
| 70 | 1.010 | 14,4 | 33,5 | 1.570 | 14400 |
| 71 | 1.020 | 14,4 | 33,9 | 1.600 | 14400 |
| 72 | 1.030 | 14,3 | 34,4 | 1.620 | 14300 |
| 73 | 1.050 | 14,3 | 34,9 | 1.640 | 14300 |
| 74 | 1.060 | 14,3 | 35,4 | 1.660 | 14300 |
| 75 | 1.080 | 14,3 | 35,8 | 1.680 | 14300 |
| 76 | 1.090 | 14,3 | 36,3 | 1.700 | 14300 |
| 77 | 1.100 | 14,3 | 36,8 | 1.730 | 14300 |
| 78 | 1.120 | 14,3 | 37,3 | 1.750 | 14300 |
| 79 | 1.130 | 14,3 | 37,8 | 1.770 | 14300 |
| 80 | 1.150 | 14,3 | 38,2 | 1.790 | 14300 |
| 81 | 1.160 | 14,3 | 38,7 | 1.810 | 14300 |
| 82 | 1.170 | 14,3 | 39,2 | 1.830 | 14300 |
| 83 | 1.190 | 14,3 | 39,7 | 1.860 | 14300 |
| 84 | 1.200 | 14,3 | 40,1 | 1.880 | 14300 |
| 85 | 1.210 | 14,3 | 40,6 | 1.900 | 14300 |
| 86 | 1.230 | 14,3 | 41,1 | 1.920 | 14300 |
| 87 | 1.240 | 14,3 | 41,6 | 1.940 | 14300 |
| 88 | 1.250 | 14,3 | 42,1 | 1.960 | 14300 |
| 89 | 1.270 | 14,3 | 42,5 | 1.990 | 14300 |
| 90 | 1.280 | 14,3 | 43 | 2.010 | 14300 |
| 91 | 1.300 | 14,3 | 43,5 | 2.030 | 14300 |
| 92 | 1.310 | 14,2 | 44 | 2.050 | 14200 |
| 93 | 1.320 | 14,2 | 44,4 | 2.070 | 14200 |
| 94 | 1.340 | 14,2 | 44,9 | 2.100 | 14200 |
| 95 | 1.350 | 14,2 | 45,4 | 2.120 | 14200 |
| 96 | 1.370 | 14,2 | 45,9 | 2.140 | 14200 |
| 97 | 1.380 | 14,2 | 46,4 | 2.160 | 14200 |
| 98 | 1.390 | 14,2 | 46,8 | 2.180 | 14200 |
| 99 | 1.410 | 14,2 | 47,3 | 2.210 | 14200 |
| 100 | 1.420 | 14,2 | 47,8 | 2.230 | 14200 |

**Table 12** Rheological data of an extra heavy crude oil at 343.15 K (70°C), 1-100 s^-1^, 0.65 T

| Shear Rate | Shear Stress | Viscosity | Speed | Torque | Viscosity |
| --- | --- | --- | --- | --- | --- |
| [1/s] | [Pa] | [Pa·s] | [1/min] | [µNm] | Cp |
| 1 | 14,8 | 14,8 | 0,478 | 23,1 | 14800 |
| 2 | 29,5 | 14,8 | 0,956 | 46,2 | 14800 |
| 3 | 44,1 | 14,7 | 1,43 | 69 | 14700 |
| 4 | 58,6 | 14,7 | 1,91 | 91,8 | 14700 |
| 5 | 73 | 14,6 | 2,39 | 114 | 14600 |
| 6 | 87,1 | 14,5 | 2,87 | 136 | 14500 |
| 7 | 101 | 14,4 | 3,34 | 158 | 14400 |
| 8 | 115 | 14,3 | 3,82 | 180 | 14300 |
| 9 | 128 | 14,2 | 4,3 | 201 | 14200 |
| 10 | 141 | 14,1 | 4,78 | 221 | 14100 |
| 11 | 154 | 14 | 5,26 | 242 | 14000 |
| 12 | 167 | 13,9 | 5,73 | 261 | 13900 |
| 13 | 179 | 13,8 | 6,21 | 281 | 13800 |
| 14 | 192 | 13,7 | 6,69 | 300 | 13700 |
| 15 | 203 | 13,6 | 7,17 | 319 | 13600 |
| 16 | 215 | 13,4 | 7,64 | 337 | 13400 |
| 17 | 227 | 13,3 | 8,12 | 355 | 13300 |
| 18 | 238 | 13,2 | 8,6 | 373 | 13200 |
| 19 | 249 | 13,1 | 9,08 | 390 | 13100 |
| 20 | 260 | 13 | 9,56 | 407 | 13000 |
| 21 | 271 | 12,9 | 10 | 424 | 12900 |
| 22 | 281 | 12,8 | 10,5 | 441 | 12800 |
| 23 | 292 | 12,7 | 11 | 457 | 12700 |
| 24 | 302 | 12,6 | 11,5 | 473 | 12600 |
| 25 | 312 | 12,5 | 11,9 | 488 | 12500 |
| 26 | 322 | 12,4 | 12,4 | 504 | 12400 |
| 27 | 331 | 12,3 | 12,9 | 518 | 12300 |
| 28 | 341 | 12,2 | 13,4 | 533 | 12200 |
| 29 | 350 | 12,1 | 13,9 | 548 | 12100 |
| 30 | 360 | 12 | 14,3 | 563 | 12000 |
| 31 | 369 | 11,9 | 14,8 | 577 | 11900 |
| 32 | 378 | 11,8 | 15,3 | 592 | 11800 |
| 33 | 387 | 11,7 | 15,8 | 606 | 11700 |
| 34 | 396 | 11,7 | 16,2 | 621 | 11700 |
| 35 | 406 | 11,6 | 16,7 | 635 | 11600 |
| 36 | 415 | 11,5 | 17,2 | 649 | 11500 |
| 37 | 423 | 11,4 | 17,7 | 663 | 11400 |
| 38 | 432 | 11,4 | 18,2 | 677 | 11400 |
| 39 | 441 | 11,3 | 18,6 | 690 | 11300 |
| 40 | 449 | 11,2 | 19,1 | 703 | 11200 |
| 41 | 459 | 11,2 | 19,6 | 719 | 11200 |
| 42 | 468 | 11,1 | 20,1 | 733 | 11100 |
| 43 | 481 | 11,2 | 20,5 | 752 | 11200 |
| 44 | 490 | 11,1 | 21 | 767 | 11100 |
| 45 | 499 | 11,1 | 21,5 | 781 | 11100 |
| 46 | 508 | 11 | 22 | 795 | 11000 |
| 47 | 516 | 11 | 22,5 | 808 | 11000 |
| 48 | 525 | 10,9 | 22,9 | 822 | 10900 |
| 49 | 534 | 10,9 | 23,4 | 836 | 10900 |
| 50 | 543 | 10,9 | 23,9 | 849 | 10900 |
| 51 | 551 | 10,8 | 24,4 | 863 | 10800 |
| 52 | 560 | 10,8 | 24,8 | 876 | 10800 |
| 53 | 568 | 10,7 | 25,3 | 889 | 10700 |
| 54 | 577 | 10,7 | 25,8 | 903 | 10700 |
| 55 | 585 | 10,6 | 26,3 | 916 | 10600 |
| 56 | 593 | 10,6 | 26,8 | 929 | 10600 |
| 57 | 602 | 10,6 | 27,2 | 942 | 10600 |
| 58 | 610 | 10,5 | 27,7 | 955 | 10500 |
| 59 | 618 | 10,5 | 28,2 | 968 | 10500 |
| 60 | 627 | 10,4 | 28,7 | 981 | 10400 |
| 61 | 635 | 10,4 | 29,1 | 994 | 10400 |
| 62 | 643 | 10,4 | 29,6 | 1.010 | 10400 |
| 63 | 654 | 10,4 | 30,1 | 1.020 | 10400 |
| 64 | 663 | 10,4 | 30,6 | 1.040 | 10400 |
| 65 | 672 | 10,3 | 31,1 | 1.050 | 10300 |
| 66 | 680 | 10,3 | 31,5 | 1.060 | 10300 |
| 67 | 688 | 10,3 | 32 | 1.080 | 10300 |
| 68 | 697 | 10,2 | 32,5 | 1.090 | 10200 |
| 69 | 705 | 10,2 | 33 | 1.100 | 10200 |
| 70 | 713 | 10,2 | 33,4 | 1.120 | 10200 |
| 71 | 721 | 10,2 | 33,9 | 1.130 | 10200 |
| 72 | 729 | 10,1 | 34,4 | 1.140 | 10100 |
| 73 | 738 | 10,1 | 34,9 | 1.160 | 10100 |
| 74 | 746 | 10,1 | 35,4 | 1.170 | 10100 |
| 75 | 754 | 10,1 | 35,8 | 1.180 | 10100 |
| 76 | 767 | 10,1 | 36,3 | 1.200 | 10100 |
| 77 | 776 | 10,1 | 36,8 | 1.210 | 10100 |
| 78 | 785 | 10,1 | 37,3 | 1.230 | 10100 |
| 79 | 795 | 10,1 | 37,7 | 1.240 | 10100 |
| 80 | 805 | 10,1 | 38,2 | 1.260 | 10100 |
| 81 | 814 | 10,1 | 38,7 | 1.270 | 10100 |
| 82 | 822 | 10 | 39,2 | 1.290 | 10000 |
| 83 | 831 | 10 | 39,7 | 1.300 | 10000 |
| 84 | 840 | 10 | 40,1 | 1.320 | 10000 |
| 85 | 851 | 10 | 40,6 | 1.330 | 10000 |
| 86 | 861 | 10 | 41,1 | 1.350 | 10000 |
| 87 | 870 | 10 | 41,6 | 1.360 | 10000 |
| 88 | 879 | 9,99 | 42 | 1.380 | 9990 |
| 89 | 887 | 9,97 | 42,5 | 1.390 | 9970 |
| 90 | 897 | 9,96 | 43 | 1.400 | 9960 |
| 91 | 906 | 9,95 | 43,5 | 1.420 | 9950 |
| 92 | 915 | 9,95 | 44 | 1.430 | 9950 |
| 93 | 926 | 9,96 | 44,4 | 1.450 | 9960 |
| 94 | 936 | 9,96 | 44,9 | 1.470 | 9960 |
| 95 | 945 | 9,95 | 45,4 | 1.480 | 9950 |
| 96 | 954 | 9,94 | 45,9 | 1.490 | 9940 |
| 97 | 963 | 9,93 | 46,3 | 1.510 | 9930 |
| 98 | 973 | 9,93 | 46,8 | 1.520 | 9930 |
| 99 | 983 | 9,93 | 47,3 | 1.540 | 9930 |
| 100 | 992 | 9,92 | 47,8 | 1.550 | 9920 |
